# Transcriptomic profiling of the salt-stress response in the wild recretohalophyte *Reaumuria trigyna*

**DOI:** 10.1186/1471-2164-14-29

**Published:** 2013-01-16

**Authors:** Zhen-hua Dang, Lin-lin Zheng, Jia Wang, Zhe Gao, Shu-biao Wu, Zhi Qi, Ying-chun Wang

**Affiliations:** 1 Key Laboratory of Herbage & Endemic Crop Biotechnology, and College of Life Sciences, Inner Mongolia University, Hohhot, 010020, PR China; 2 School of Environmental and Rural Science, University of New England, Armidale, NSW, 2351, Australia

**Keywords:** *Reaumuria trigyna*, Recretohalophyte, Transcriptome, Illumina sequencing, Salt-stress response

## Abstract

**Background:**

*Reaumuria trigyna* is an endangered small shrub endemic to desert regions in Inner Mongolia. This dicotyledonous recretohalophyte has unique morphological characteristics that allow it to tolerate the stress imposed by semi-desert saline soil. However, it is impossible to explore the mechanisms underlying this tolerance without detailed genomic information. Fortunately, newly developed high-throughput sequencing technologies are powerful tools for *de novo* sequencing to gain such information for this species.

**Results:**

Two sequencing libraries prepared from control (C21) and NaCl-treated samples (T43) were sequenced using short reads sequencing technology (Illumina) to investigate changes in the *R. trigyna* transcriptome in response to salt stress. Among 65340 unigenes, 35495 (52.27%) were annotated with gene descriptions, conserved domains, gene ontology terms, and metabolic pathways with a cut-off E-value of 10^-5^. These included 44 Gene Ontology (GO) terms, 119 Kyoto Encyclopedia of Genes and Genomes (KEGG) pathways, and 25 Clusters of Orthologous Groups families. By comparing the transcriptomes from control and NaCl-treated plants, 5032 genes showed significantly differences in transcript abundance under salt stress (false discovery rate ≤ 0.001 and |log_2_Ratio| ≥ 1). These genes were significantly enriched in 29 KEGG pathways and 26 GO terms. The transcription profiles indicated that genes related to ion transport and the reactive oxygen species scavenging system were relevant to the morphological and physiological characteristics of this species. The expression patterns of 30 randomly selected genes resulted from quantitative real-time PCR were basically consistent with their transcript abundance changes identified by RNA-seq.

**Conclusions:**

The present study identified potential genes involved in salt tolerance of *R. trigyna*. The globally sequenced genes covered a considerable proportion of the *R. trigyna* transcriptome. These data represent a genetic resource for the discovery of genes related to salt tolerance in this species, and may be a useful source of reference sequences for closely related taxa. These results can also further our understanding of salt tolerance in other halophytes surviving under sodic stress.

## Background

Soil salinity is one of the most significant abiotic stresses limiting plant growth, productivity, and geographical distribution [1,2]. Approximately 20% of the world’s cultivated lands, and nearly 50% of irrigated lands, are affected by salinity [3,4]. High salt levels impose two stresses on plants: osmotic stress, resulting from the lowered water availability because of the high osmotic pressure in the soil; and ionic stress arising from solute imbalance, in which there are increased levels of Na^+^ and Cl^-^ in the cytosol and changes to the intracellular K^+^/Na^+^ ratio [5,6]. Halophytes are the natural inhabitants of highly saline soils, and have evolved resistance strategies including efficient control of the uptake and compartmentalization of salt ions, synthesis of organic ‘compatible’ solutes, and unique morphological structures such as succulent leaves, salt glands, and bladders [7]. Such remarkable adaptations to salty soil make halophytes excellent candidates to investigate salt-tolerance mechanisms and to identify effective salt-response genes in this type of flora [8].

Studies on the salt-tolerance mechanisms of halophytes have been carried out recently in several model plants, such as *Thellungiella halophila*, *Mesembryanthemum crystallinum*, *Suaeda*, and *Populus*. Excellent progress has been made in understanding the physiological and molecular mechanisms underlying stress tolerance. However, knowledge about halophytes and their physiology is rather limited with respect to their wide taxonomic occurrence. It remains unclear whether halophytes have unique tolerance mechanisms, and whether such mechanisms are taxonomically linked, and/or have evolved to respond to interactions between salinity and other environmental variables. Therefore, studies on several species from different salt-stressed environments should be performed to answer these questions [9].

*Reaumuria trigyna* (*Reaumuria* Linn genus, family Tamaricaceae) is an endangered dicotyledonous shrub with the features of a recretohalophyte. It is regarded as a living fossil owing to its Tethys Ocean origin [10-13]. This species is endemic to the Eastern Alxa–Western Ordos area (106°27’E–111°28’E, 39°13’N–40°52’N, elevation 1500–2100 m), a salinized desert in Inner Mongolia, China. The area is characterized by its high soil salinity (up to 0.7% salts), hyper-drought conditions (annual average precipitation of 140.9–302.2 mm), and low temperature (annual average temperature of 6.0–9.2°C) [14]. To adapt to the semi-desert and salty soil environment, *R. trigyna* has developed a distinct morphology that is characterized by succulent leaves and sunken stomata. These features are typical of a recretohalophyte—a halophyte with salt excretion glands. According to our previous studies, the concentrations of Na^+^, Cl^-^, and SO_4_^2-^ in the soil where *R. trigyna* grows are higher than those of K^+^, Mg^2+^, and Ca^2+^. The Na^+^ concentration in the soil is as high as 0.6 mg/g, with a Na^+^/K^+^ ratio of approximately 16. *R. trigyna* excretes Na^+^ and Cl^-^ via its multicellular salt glands, but little K^+^ is excreted, with a Na^+^/K^+^ ratio of only 0.44. When seedlings were treated with 400 mM NaCl, *R. trigyna* showed a rapid increase in glutathione (GSH) from nearly 4.77 to 9.62 nmol·g^-1^ FW, and showed significant increases in the activities of superoxide dismutase (SOD) and peroxidase (POD) (*P* < 0.05) [15]. As a typical recretohalophyte growing in this area, *R. trigyna* could be considered as a reference species to represent those that survive in the severely adverse environment of the Ordos desert. Knowledge about the unique adaption strategies employed by *R. trigyna* will provide valuable references to the salt-tolerance mechanisms in these species and possibly in other halophytes growing in different environments. However, these objectives are difficult to achieve without detailed genetic and sequence information for this species. Fortunately, powerful high throughput sequencing technologies are now available to sequence the genome of plants *de novo*. Therefore, it is now possible to obtain the required sequences of *R. trigyna* to address important questions about its physiology, such as its mechanisms of salt-tolerance.

Next-generation sequencing (NGS) technology has developed rapidly in recent years. It not only provides rapid, cost-effective, and comprehensive analyses of complex nucleic acid populations for model plants or species closely related to model plants, but also provides opportunities to analyze non-model plants whose genomes have never been sequenced [16-19]. This technology has been used widely in comparative transcriptomics to identify differences in transcript abundance among different cultivars, organs, and different treatment conditions [20-22]. In the present study, we generated a transcriptome dataset to provide genetic information to explore the salt-tolerance mechanisms of *R. trigyna* using the Illumina HiSeq™ 2000 platform. We compared transcriptomes of salt-stressed and control plants to identify genes showing transcriptional changes, and identified the functions of the transcripts and the KEGG pathways that showed changes. We speculate that the assembled, annotated transcriptome sequences and transcript abundance patterns will provide a valuable genetic resource for further investigations of the molecular mechanisms of salt tolerance in this species, and possibly in other recretohalophytes.

## Results

### Sequencing output and assembly

**Figure 1:**
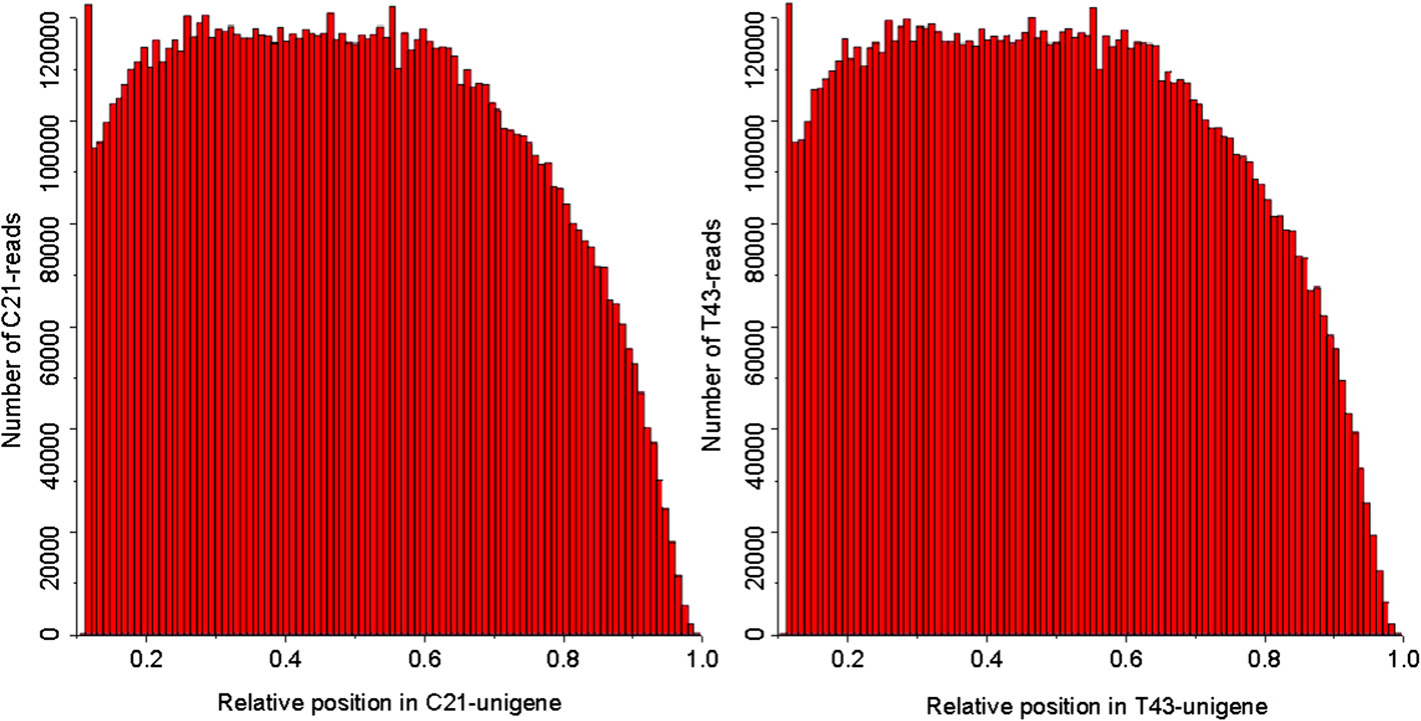
**Random distribution of sequencing reads in assembled unigenes.** X-axis represents relative position of sequencing reads in the assembled sequences. Orientation of assembled unigenes is from the 5’ end to the 3’end. Y-axis indicates number of reads. Left: random distribution of C21 reads mapped to C21-unigenes. Right: random distribution of T43 reads mapped to T43-unigenes.

Two sequencing libraries were prepared from control (C21) and NaCl-treated samples (T43) to investigate the transcriptomic responses to salt-stress in *R. trigyna*. In total, 26.51 million raw reads were generated from C21 and 28.17 million raw reads were generated from T43 (Sequence Read Archive accession number: SRP008320; Table 1). The raw reads had an average length of 90 bp. Among all the raw reads, more than 92% had Phred-like quality scores at the Q20 level (an error probability of 1%). These were used for *de novo* assembly. As a result, 459601 (C21) and 476229 (T43) contigs, and 97752 (C21) and 101931 (T43) scaffolds, were generated. After removal of redundancy, 68076 (C21) and 71194 (T43) unigenes were clustered using the Gene Indices Clustering Tools (GICT) program (Table 1, deposited in the Transcriptome Shotgun Assembly Sequence database. Accession numbers are summarized in Additional files 1 and 2). The random distribution of reads in the above-assembled unigenes indicated that the 3’ ends of all assembled unigenes contained relatively fewer reads compared with other positions within these unigenes, which showed greater and more even distributions (Figure 1).

**Table 1:** Summary of sequencing and assembly results

	**C21 (number)**	**Mean length (bp)**	**N50 (bp)**	**T43 (number)**	**Mean length (bp)**	**N50 (bp)**
Raw reads	26512992			28166582		
Clean reads	25555558			27266668		
Contigs	459601	138	107	476229	138	106
Scaffolds	97752	341	475	101931	337	463
Unigenes	68076	559	437	71194	541	429
All-unigene	Number: 65340		Mean length (bp): 770		N50 (bp): 562	

Mean length: Mean length of assembled sequences.

N50: 50% of the assembled bases were incorporated into sequences with length of N50 or longer.

**Figure 2:**
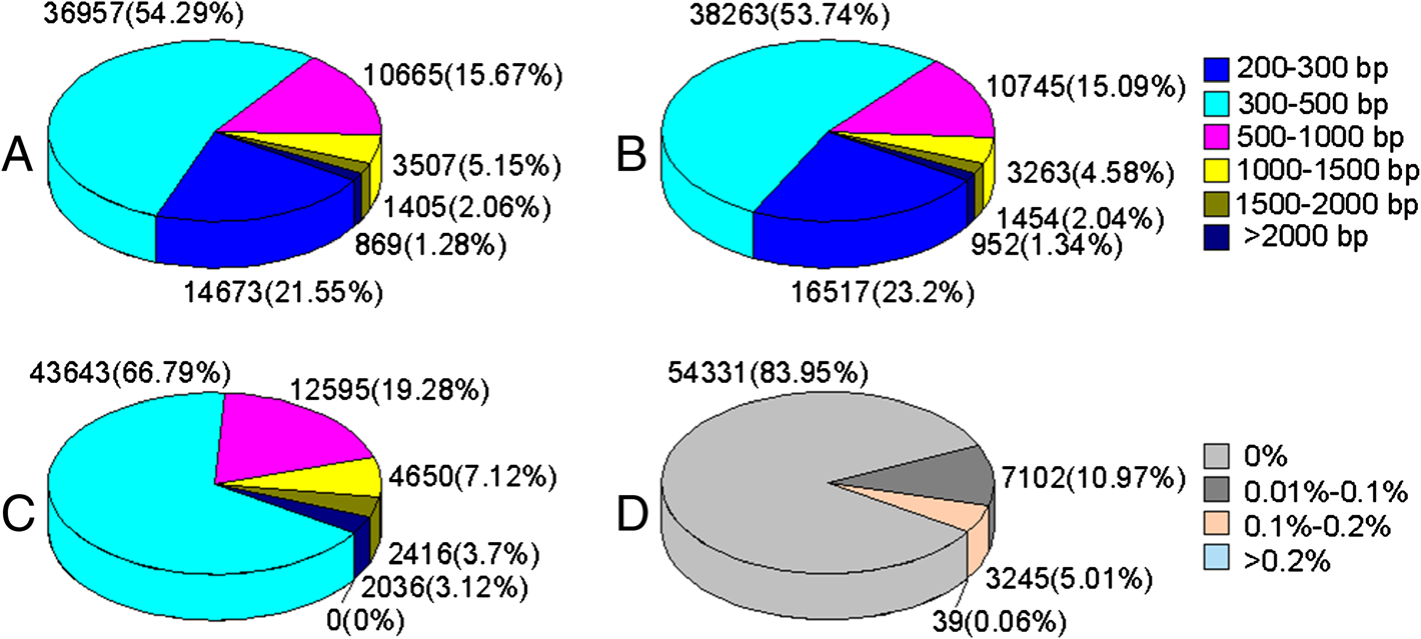
**Length distribution of C21-, T43- and all-unigenes, and gap distribution of all unigenes. A, B, C:** Length distribution of C21-unigenes, T43-unigenes, and all-unigenes. Values in colored square brackets indicate range of unigene length. Unigene numbers and percentages (total number of unigenes in a certain length range) are shown next to pie chart. Total number of C21-, T43-, and all-unigenes was 68076, 71194, and 65340, respectively. **D:** Gap distribution of all unigenes. Numbers in square brackets represent ratio of N number for one unigene.

Using the CAP3 assembler, we obtained 65340 all-unigenes with an average length of 562 bp and an N50 of 770 bp by combining C21 (25.6 million) and T43 (27.3 million) clean reads (Table 1). Of the 65340 unigenes, 34745 were clustered from the assembled C21 (43506) and T43 (44558) unigenes. The remaining 30595 sequences were singletons with 14764 from C21 and 15831 from T43 unigenes. The length distributions of the unigenes from both C21 and T43 were similar with sequences in the 200–300 bp range making up 21.55% and 23.2% of C21 and T43 unigenes, respectively. The length of all-unigenes was dramatically increased following further assembly of the C21 and T43 unigenes, resulting in assembled sequences with lengths of > 300 bp (Figure 2). Ultimately, 54331 gap-free sequences were obtained by assembling all of the C21- and T43-unigenes.

### Functional annotation of assembled unigenes

**Figure 3:**
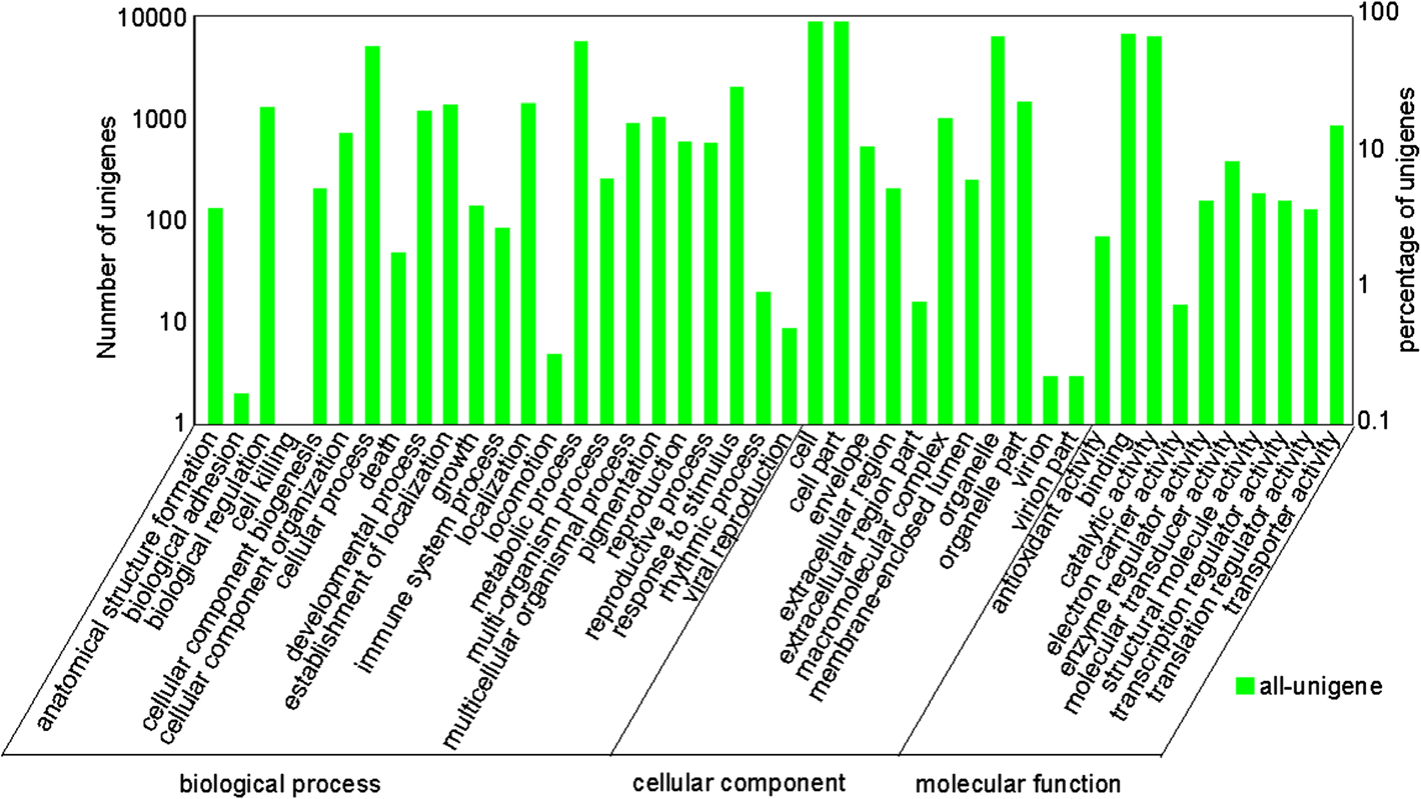
**GO functional annotation.** Unigenes with best BLAST hits were aligned to GO database. All 13552 unigenes were assigned to at least one GO term and were grouped into three main GO categories and 44 sub-categories. Right Y-axis represents number of genes in a category. Left Y-axis indicates percentage of a specific category of genes in each main category.

All assembled high-quality unigenes were first blasted against the NCBI non-redundant (nr) database using BLASTX with a cut-off E-value of 10^-5^. Of the 65340 all-unigenes, 35271 (53.9%) returned at least one match at the E-value > 10^-5^. Because of the lack of genome and EST information for *R. trigyna* or closely related taxa, 46.2% of the unigenes did not match to known genes in the database. Based on sequence homology, 13552 unigenes were categorized into 44 Gene Ontology (GO) terms (Figure 3, Additional file 3). In each of the three main GO classifications, i.e., biological process, cellular component, and molecular function, “cellular process”, “metabolic process”, “cell”, “cell part” and “binding” terms were dominant among the returned terms. Many of the identified unigenes were classified in the categories “response to stimulus”, “organelle” and “catalytic activity”, whereas only a few genes belonged to “biological adhesion”, “cell killing”, “locomotion”, “viral reproduction”, “virion” and “virion part”. Interestingly, 894 unigenes were classified into the category “transporter activity” and 71 unigenes into the category “antioxidant activity”.

**Figure 4:**
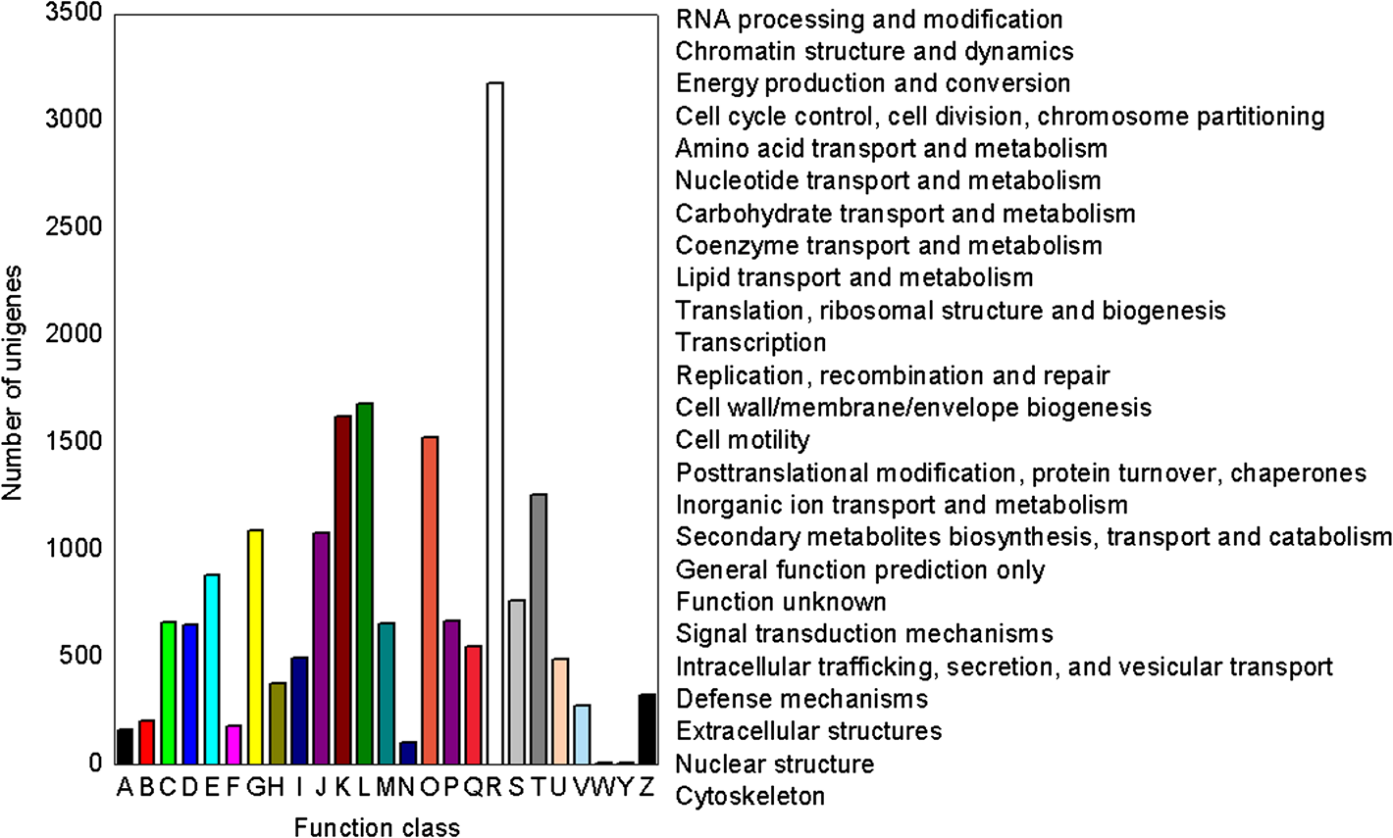
**COG classification of putative proteins.** All 10968 putative proteins showing significant homology to those in COG database were functionally classified into 25 molecular families. Y-axis indicates number of unigenes in a specific functional cluster.

To further evaluate the integrity of our transcriptome library and the effectiveness of our annotation process, unigene sequences were subjected to Clusters of Orthologous Groups (COG) classification. Out of 35271 nr hits, 10968 sequences showed a COG classification (Figure 4, Additional file 3). Among the 25 COG categories, the cluster for “general function prediction only” (3179, 28.98%) was the largest group, followed by “replication, recombination and repair” (1681, 15.32%), “transcription” (1621, 14.78%), “signal transduction mechanisms” (1257, 11.46%), and “carbohydrate transport and metabolism” (1089, 9.93%). The categories “nuclear structure” (8, 0.07%) and “extracellular structures” (7, 0.06%) had the fewest corresponding genes.

The 35271 annotated sequences were mapped to the reference canonical pathways in the Kyoto Encyclopedia of Genes and Genomes (KEGG). Among those, 15995 unigenes were assigned to 119 KEGG pathways (Additional file 3). The pathways most strongly represented by mapped unigenes were “metabolic pathways” (3459 unigenes), “biosynthesis of secondary metabolites” (1920 unigenes), and “plant-pathogen interaction” (1110 unigenes), indicating that these were the active pathways in *R. trigyna*. Finally, 38197 unigenes were annotated in the transcriptome of *R. trigyna*, of which 35459 were generated by BLAST analysis and 2738 were annotated using the ESTscan program.

### Identification and annotation of potential differentially expressed genes (DEGs)

**Figure 5:**
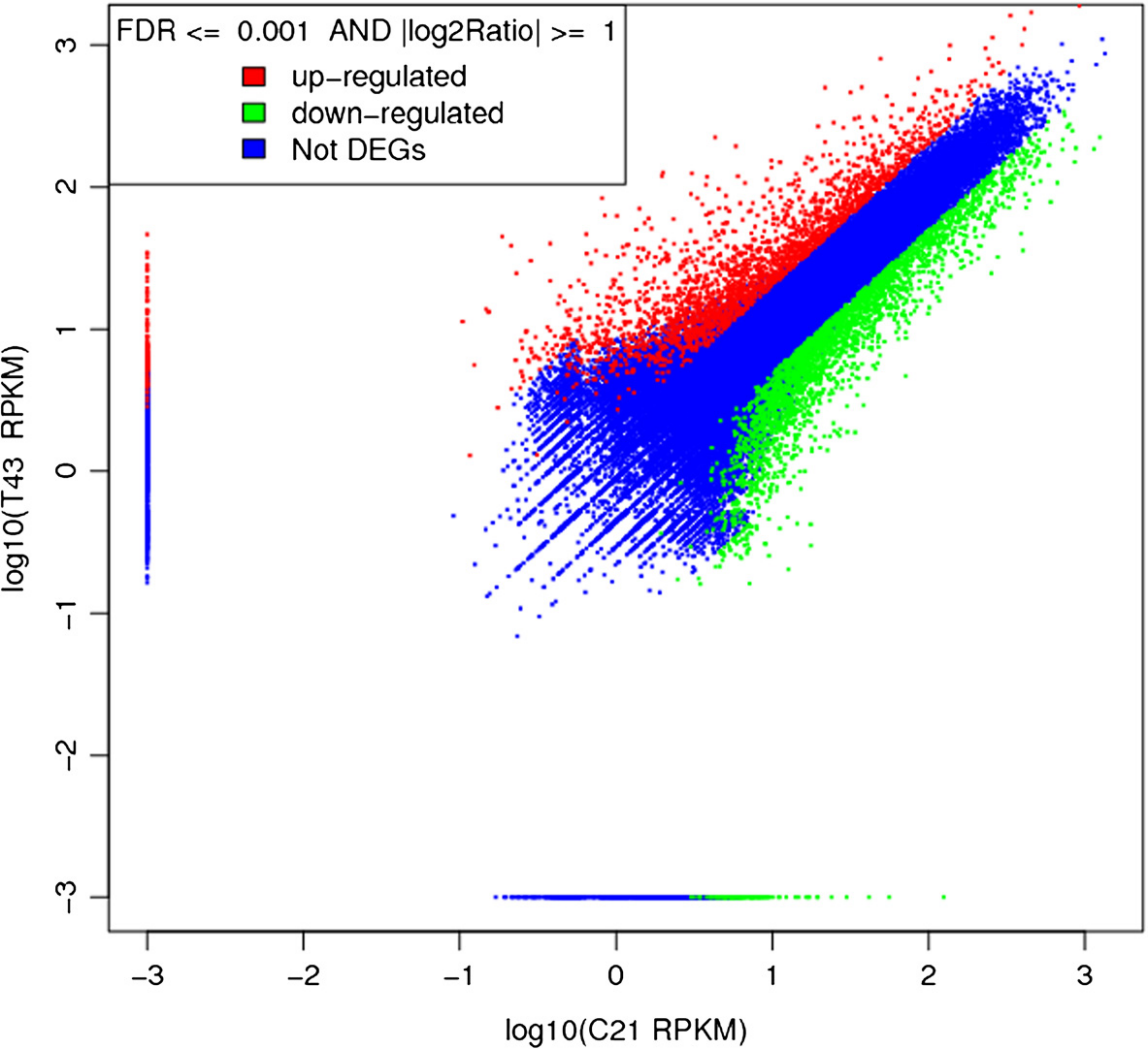
**Identification of DEGs between C21 and T43.** DEGs were determined using a threshold of FDR ≤ 0.001 and |log_2_Ratio| ≥ 1. Red spots represent up-regulated DEGs and green spots indicate down-regulated DEGs. Those shown in blue are unigenes that did not show obvious changes in salt-stressed *R. trigyna*.

The analysis showed that 32697 unigenes were up-regulated and 31997 were down-regulated upon treatment of *R. trigyna* seedlings with salt solution. In total, 5032 DEGs were identified in both libraries, including 2370 up- and 2662 down-regulated unigenes (Figure 5). Among those DEGs responding to salt-stress treatment, 3719 were annotated and 1313 had no BLAST hits.

**Figure 6:**
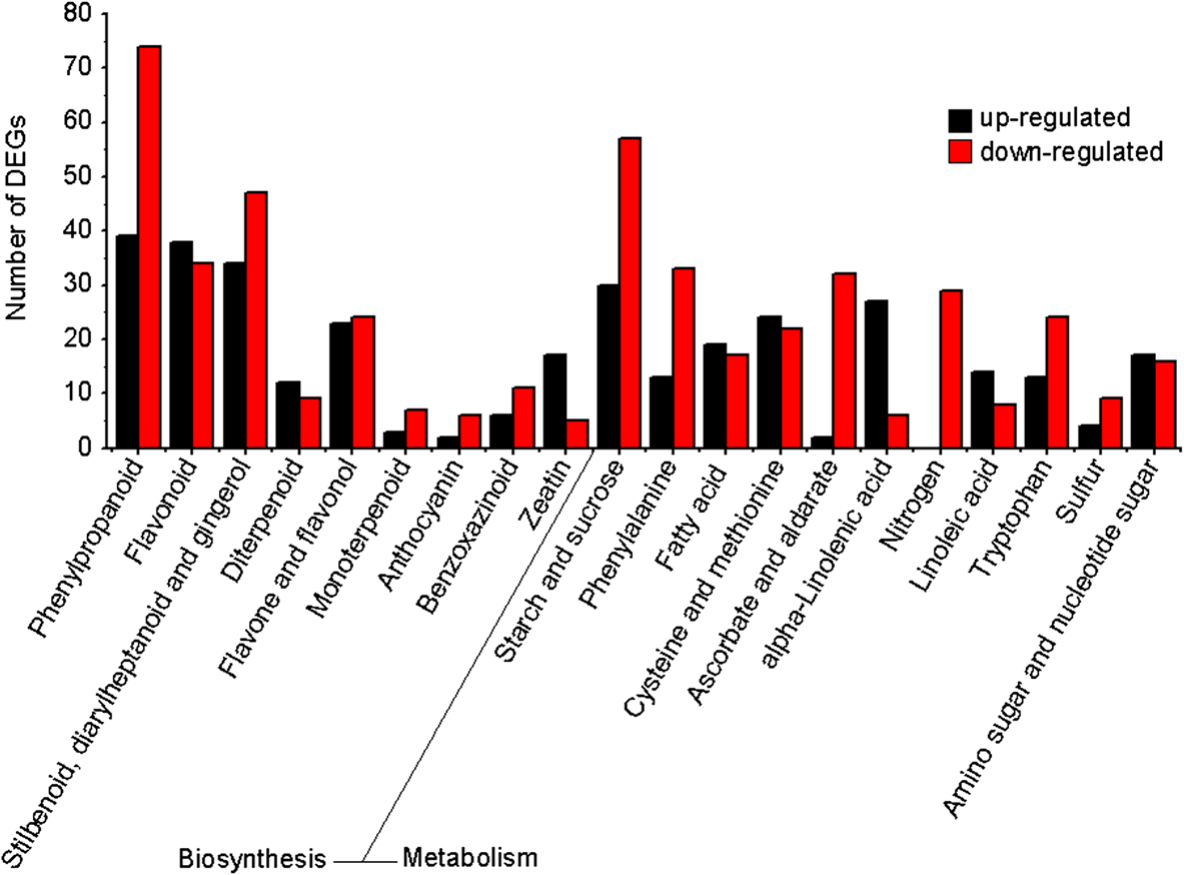
**KEGG enrichment analysis showed significantly enriched biosynthesis and metabolic pathways.** Y-axis indicates numbers of DEGs enriched in KEGG pathways. X-axis represents DEGs enriched in secondary metabolites biosynthesis and metabolism pathways. Black bar represents up-regulated DEGs; red bar indicates down-regulated DEGs.

Enrichment analysis was conducted to clarify the biological functions of the identified DEGs. The results indicated that 1947 DEGs were enriched in 27 GO terms, using a corrected P-value ≤ 0.05 as the threshold (Additional file 4). Among these GO categories, “oxidoreductase activity” (171 DEGs), “catalytic activity” (742 DEGs) and “response to stress” (155 DEGs) were significantly enriched among DEGs compared with the whole transcriptome background. In total, 2086 DEGs were enriched in 33 metabolic pathways (q-value ≤ 0.05) (Additional file 4). The most enriched metabolic pathways were “metabolic pathways” (483 DEGs), “biosynthesis of secondary metabolites” (351 DEGs), and “plant-pathogen interaction” (198 DEGs). The pathways of phenylpropanoid biosynthesis, flavonoid biosynthesis, stilbenoid, diarylheptanoid, and gingerol biosynthesis were very active, while those associated with ascorbate, aldarate, and nitrogen metabolism were strongly suppressed in salt-treated plants (Figure 6, Additional file 5).

### Genes related to ion transport

**Figure 7:**
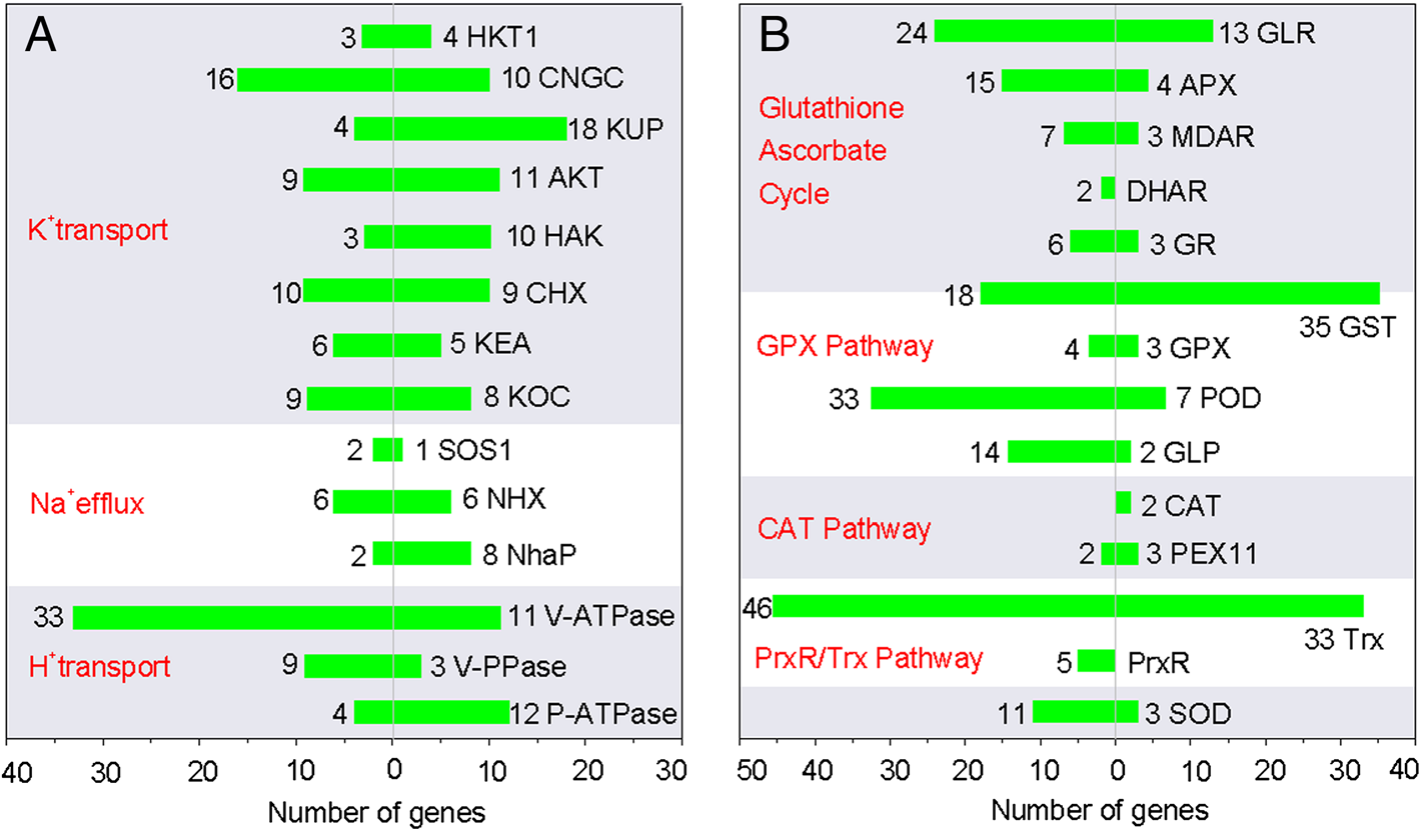
**Transcripts related to ion transport and ROS scavenging system.** Columns at left of Y-axis show genes with down-regulated transcription under salt stress; columns at right of Y-axis show genes with up-regulated transcription under salt stress. Gene numbers and categories are shown next to columns. **A:** Transcriptional characteristics of genes related to ion transport. HKT, high-affinity K^+^ transporter; CNGC, cyclic nucleotide-gated channel; KUP, K^+^ ion transmembrane transporter; AKT, K^+^ channel; HAK, high affinity K^+^ transporter; CHX, cation H^+^ exchanger; KEA, K^+^ efflux antiporter; KOC, outward rectifying K^+^ channel; SOS1, salt overly sensitive 1; NHX, Na^+^/H^+^ exchanger; NhaP, NhaP-type Na^+^/H^+^ antiporter; V-ATPase, V-H^+^-ATPase; V-PPase, V-H^+^-PPase; P-ATPase, PM-H^+^-ATPase. **B:** Transcriptional characteristics of genes related to ROS scavenging system. GLR, glutaredoxin; APX, ascorbate peroxidase; MDAR, monodehydroascorbate reductase; DHAR, dehydroascorbate reductase; GR, glutathione reductase; GST, glutathione S-transferase; GPX glutathione peroxidase; POD, peroxidases; GLP, germin-like protein; CAT, catalase; PEX11, peroxisomal biogenesis factor11; Trx, thioredoxin; PrxR, peroxiredoxin; SOD, superoxide dismutase. GST is used in both the glutathione ascorbate cycle and GPX pathway.

Among the unigenes, we identified 135, 72, and 25 genes related to regulation of K^+^ uptake, H^+^ pumping, and Na^+^ efflux, respectively (Figure 7, Table 2, Additional file 6). Clearly, genes associated with K^+^-transport formed the largest proportions of screened genes, suggesting that these genes played important role in reestablishing Na^+^/K^+^ homeostasis in salt-stressed *R. trigyna*. Among 135 K^+^ transport genes, 42 were DEGs, including 24 up- and 18 down-regulated genes in response to salt-stress. KUP (unigene14170, unigene26051) and CNGC (unigene3965, unigene7183) transcripts were present at high levels in both libraries, but were down-regulated by nearly 2-fold under salt-stress. Seven HKT1 genes, which are responsible for Na^+^ influx, were salt-responsive, of which three (unigene4890, unigene27586, and unigene20631) were suppressed by more than 2-fold. We identified 16, 44, and 12 genes encoding plasma membrane H^+^-ATPases (PM-H^+^-ATPases), vacuolar H^+^-ATPases (V-H^+^-ATPases) and H^+^-pyrophosphatases (V-H^+^-PPases), respectively. Out of 44 V-ATPase homologs, four showed down-regulated transcription under salt treatment while the other 40 did not. Transcripts of six PM-H^+^-ATPase homologs were highly abundant in both libraries. Two PM-H^+^-ATPases (unigene15650 and unigene248) were up-regulated by salt, showing increases in their Reads Per Kilobase of exon model per Million mapped reads (RPKM) values from 143.1 to 376.9 and from 79.8 to 240.3, respectively. Five PPases showed high transcript levels in both libraries, but their transcriptions did not change under salt-stress. Among 35 Na^+^ efflux genes, one PM-Na^+^/H^+^ antiporter gene (unigene798 or SOS1B) showed moderate transcript levels and two showed low transcript abundance. Transcription of unigene798 was slightly up-regulated by salt-stress (0.3-fold increase in abundance). Among 12 genes encoding V-Na^+^/H^+^ antiporter family proteins, four (unigene20634, unigene5272, unigene8445, and unigene752) were highly abundant. The transcript levels of unigene20634 and unigene5272 were up-regulated by nearly 2-fold, whereas those of unigene8445 and unigene752 were unchanged under salt-stress. There were abundant *Nha* (multisubunit Na^+^/H^+^ antiporter, unigene16859) transcripts in both libraries (RPKM values of 223.0 in C21 and 311.2 in T43).

**Table 2:** DEGs and highly transcribed genes related to ion transport *

**GeneID**	**C21 RPKM**	**T43 RPKM**	**Fold change**	**Homologous species**	**GeneID**	**C21 RPKM**	**T43 RPKM**	**Fold change**	**Homologous species**
**HKT1**					**SOS**				
Ug20631	28.6	9.5	−1.6	*P. trichocarpa*	Ug798	27.2	34.5	+0.3	*C. quinoa*
Ug4890	139.4	64.2	−1.1	*P. trichocarpa*	Ug35995	0.5	0.0	−8.9	*C. quinoa*
Ug27586	70.8	30.8	−1.2	*M. crystallinum*	Ug35827	3.4	0.9	−1.9	*M. crystallinum*
**CNGC**					**NHX**				
Ug3965	141.8	101.0	−0.5	*A. thaliana*	Ug20634	23.2	44.6	+0.9	*C. reticulata*
Ug7183	112.6	86.2	−0.4	*G. hirsutum*	Ug5272	10.1	23.7	+1.2	*S. komarovii*
Ug24497	9.8	4.6	−1.1	*A. lyrata*	Ug8445	47.3	39.3	−0.3	*M. crystallinum*
Ug61678	0.0	3.2	+11.7	*A. lyrata*	Ug752	28.7	21.7	−0.4	*T. tetragonioides*
Ug54762	0.0	0.8	+9.6	*A. lyrata*	**Nha-like protein**				
Ug39788	1.3	0.0	−10.3	*V. vinifera*	Ug16859	223.0	311.2	+0.5	*T. octandrum*
**KUP**					**V-H**^**+**^**-ATPase**				
Ug14170	231.6	124.1	−0.9	*V. vinifera*	Ug63112	0.0	0.3	+8.4	*A. lyrata*
Ug26051	103.6	63.8	−0.7	*M. crystallinum*	Ug15764	0.0	0.3	+8.4	*A. lyrata*
Ug41960	15.6	33.5	+1.1	*G. hirsutum*	Ug13159	12.2	23.7	+1.0	*G. max*
Ug65296	3.3	13.7	+2.0	*G. hirsutum*	Ug8615	69.1	112.3	+0.7	*C. sinensis*
Ug61613	3.6	13.3	+1.9	*G. hirsutum*	Ug32194	16.8	24.8	+0.6	*G. max*
Ug29610	4.9	10.5	+1.1	*V. vinifera*	Ug14517	149.2	193.3	+0.4	*C. limon*
Ug5469	3.3	10.1	+1.6	*A. thaliana*	Ug48766	86.3	108.1	+0.3	*E. guineensis*
Ug33474	3.3	9.9	+1.6	*A. thaliana*	Ug23403	16.4	20.4	+0.3	*A. lyrata*
Ug4060	5.4	2.0	−1.5	*A. thaliana*	Ug27100	86.4	106.0	+0.3	*A. lyrata*
Ug41099	4.2	0.7	−2.5	*A. thaliana*	Ug5921	64.1	78.6	+0.3	*A. lyrata*
**AKT**					Ug38012	38.0	46.3	+0.3	*Z. mays*
Ug20360	3.3	7.0	+1.1	*P. tenuiflora*	Ug18074	52.5	63.8	+0.3	*C. unshiu*
Ug57424	0.0	5.6	+12.5	*P. euphratica*	Ug5444	35.8	41.4	+0.2	*D. carota*
Ug63704	3.9	7.8	+1.0	*E. camaldulensis*	Ug8331	96.0	104.3	+0.1	*E. guineensis*
Ug63924	1.0	5.9	+2.6	*V. vinifera*	Ug6864	97.1	104.6	+0.1	*G. hirsutum*
Ug47159	3.0	0.8	−2.0	*S. tuberosum*	Ug14833	77.6	83.3	+0.1	*Z. mays*
Ug4060	5.4	2.0	−1.5	*A. thaliana*	Ug32662	113.7	119.2	+0.1	*Z. mays*
Ug41099	4.2	0.7	−2.5	*A. thaliana*	Ug18679	58.5	59.7	+0.0	*A. thaliana*
**HAK**					Ug14939	58.5	59.7	+0.0	*A. thaliana*
Ug27946	36.3	74.8	+1.0	*M. crystallinum*	Ug13111	50.8	51.3	+0.0	*H. caspica*
Ug28118	13.8	31.4	+1.2	*A. thaliana*	Ug27358	137.8	138.0	+0.0	*C. unshiu*
Ug65080	3.2	8.0	+1.3	*O. sativa*	Ug31534	107.4	106.3	+0.0	*A. thaliana*
Ug54929	1.9	7.0	+1.9	*O. sativa*	Ug31417	116.0	114.7	+0.0	*K. foliatum*
Ug64325	1.5	6.1	+2.0	*V. vinifera*	Ug12019	68.3	67.4	+0.0	*G. hirsutum*
Ug35034	2.0	4.3	+1.1	*O. sativa*	Ug46409	15.4	14.6	−0.1	*Z. mays*
Ug64584	0.0	4.2	+12.1	*O. sativa*	Ug31885	133.0	124.2	−0.1	*S. salsa*
Ug48231	9.3	2.5	−1.9	*H. vulgare*	Ug9415	7.9	7.3	−0.1	*A. lyrata*
**CHX**					Ug30783	220.6	202.0	−0.1	*Z. marina*
Ug64193	2.2	6.1	+1.5	*P. trichocarpa*	Ug9762	54.3	48.2	−0.2	*A. lyrata*
Ug64762	0.0	5.3	+12.4	*P. trichocarpa*	Ug23822	289.1	241.7	−0.3	*P. acutifolius*
Ug22739	10.4	4.8	−1.1	*A. thaliana*	Ug28038	166.2	135.7	−0.3	*S. oleracea*
Ug62951	6.4	2.5	−1.4	*M. truncatula*	Ug14408	57.5	45.4	−0.3	*G. hirsutum*
Ug47929	7.6	1.0	−2.9	*A. thaliana*	Ug1235	57.5	45.4	−0.3	*G. hirsutum*
Ug47939	2.7	1.0	−1.4	*A. thaliana*	Ug14963	116.2	87.3	−0.4	*M. crystallinum*
Ug35886	1.1	0.5	−1.2	*P. trichocarpa*	Ug27790	18.8	14.1	−0.4	*A. thaliana*
**KEA**					Ug58281	139.3	103.6	−0.4	*I. lactea*
Ug39703	3.0	0.8	−2.0	*A. thaliana*	Ug62214	45.5	32.7	−0.5	*A. lyrata*
Ug43622	5.5	2.4	−1.2	*A. thaliana*	Ug3474	8.4	5.9	−0.5	*O. sativa*
Ug53119	3.5	8.9	+1.3	*A. thaliana*	Ug27883	47.2	33.0	−0.5	*M. truncatula*
Ug54277	2.6	7.3	+1.5	*A. thaliana*	Ug26948	62.4	36.4	−0.8	*G. hirsutum*
**KOC**					**PM-H**^**+**^**-ATPase**				
Ug23367	3.0	6.4	+1.1	*N. tabacum*	Ug248	79.8	240.3	+1.6	*D. carota*
Ug37885	2.4	0.8	−1.6	*H. brasiliensis*	Ug15650	143.1	376.9	+1.4	*P. persica*
Ug43016	1.7	0.5	−1.8	*--*	Ug1087	171.3	265.1	+0.6	*S. lycopersicum*
Ug19269	6.7	12.6	+0.9	*P. trichocarpa*	Ug47575	285.8	402.4	+0.5	*C. sativus*
Ug8178	9.1	10.3	+0.2	*P. trichocarpa*	Ug7564	205.5	243.9	+0.2	*A. lyrata*
Ug56865	6.4	8.3	+0.4	*P. trichocarpa*	Ug24901	199.6	218.9	+0.1	*A. thaliana*
Ug9256	32.1	55.3	+0.8	*P. trichocarpa*	**V-H**^**+**^**-PPase**				
Ug20095	7.3	7.8	+0.1	*P. trichocarpa*	Ug12515	188.6	156.3	−0.3	*A. lyrata*
Ug22675	44.6	25.4	−0.8	*P. trichocarpa*	Ug14495	251.2	184.2	−0.4	*P. communis*
Ug40263	4.4	3.4	−0.4	*P. trichocarpa*	Ug16866	276.0	268.2	+0.0	*N. tabacum*
Ug50188	3.7	3.5	−0.1	*P. trichocarpa*	Ug61784	320.3	237.0	−0.4	*V. radiata*
Ug39519	0.9	0.5	−0.9	*P. trichocarpa*	Ug8875	197.0	221.1	+0.2	*M. truncatula*
Ug25217	20.7	18.6	−0.2	*P. trichocarpa*					

*DEG was filtered using threshold of false discovery rate (FDR) ≤ 0.001 and absolute value of log_2_Ratio ≥ 1. Ug represents unigene. RPKM indicates RPKM values of unigenes in C21 or T43. “Fold change” equals to log_2_ (T43-RPKM/C21-RPKM). “+” indicates up-regulated transcription and “-” represents down-regulated transcription. Homologous species is that identified from BLAST search of nr database using the cut-off E-value of ≤ 10^-5^.

### Genes related to the ROS scavenging system

In total, 298 unigenes were predicted to encode enzymes related to reactive oxygen species (ROS) scavenging (Table 3, Additional file 7). There were 130, 84, 63, and 7 unigenes categorized into the GSH-ascorbate cycle, the peroxiredoxin/thioredoxin (PrxR/Trx) pathway, the glutathione peroxidase (GPX) pathway, and the catalase (CAT) pathway, respectively. The largest group was genes encoding thioredoxins (Trxs), glutathione S-transferases (GSTs), glutaredoxins (GLRs), and PODs (Figure 7). We detected 14 genes encoding SODs; three were up-regulated and 11 were down-regulated by salt treatment. In the GSH-ascorbate ROS removal pathway, seven ascorbate peroxidase (APX) genes (unigene44406, unigene47495, unigene17786, unigene13095, unigene57465, unigene25879, unigene10954), two GLR genes (unigene33573, unigene7889), one monodehydroascorbate reductase gene (unigene33304), one dehydroascorbate reductase gene (unigene42162), and two glutathione reductase genes (unigene8218, unigene3408) showed relatively abundant transcript levels (RPKM values of > 200). We identified five PrxRs, which play a role in the PrxR/Trx pathway in the antioxidant defense system. Two PrxR genes (unigene63275 and unigene61053) showed high transcript levels in both control and salt-treated plants (RPKM values of > 200). Out of 79 Trxs, five transcripts (unigene12493, unigene17142, unigene7940, unigene34328, and unigene2658) were expressed at high levels in both libraries. In the GPX pathway, there were 7 unigenes encoding GPXs but 53 unigenes encoding GSTs. Ten GST showed increased transcription under salt-stress, with RPKM values ranging from 102.6 to 477.2 in the T43 library but from 62.6 to 313.4 in the C21 library. In addition, 11 genes belonging to the *Tau* family of GSTs showed increased transcription under salt stress.

**Table 3:** DEGs and highly transcribed genes related to ROS scavenging system

**GeneID**	**C21 RPKM**	**T43 RPKM**	**Fold change**	**Homologous species**	**GeneID**	**C21 RPKM**	**T43 RPKM**	**Fold change**	**Homologous species**
**GLR**					Ug30784	2.2	4.9	+1.1	*P. trichocarpa*
Ug33573	239.7	239.4	+0.0	*S. tuberosum*	Ug39822	1.6	4.2	+1.4	*G. max*
Ug7889	215.9	207.0	−0.1	*P. trichocarpa*	Ug46671	3.1	4.0	+0.3	*P. trichocarpa*
Ug5332	224.8	159.1	−0.5	*A. lyrata*	Ug34203	5.5	3.9	−0.5	*P. trichocarpa*
Ug20320	59.6	137.6	+1.2	*P. trichocarpa*	Ug39974	0.9	3.8	+2.0	*C. chinense*
Ug2849	17.2	48.0	+1.5	*P. trichocarpa*	Ug49935	0.3	3.1	+3.3	*P. trichocarpa*
Ug64057	0.6	46.6	+6.2	*R. australe*	Ug49935	0.3	3.1	+3.3	*P. trichocarpa*
Ug6417	6.5	28.5	+2.1	*P. trichocarpa*	Ug51894	1.1	2.9	+1.4	*P. juliflora*
Ug64851	5.1	26.3	+2.4	*P. trichocarpa*	Ug55860	0.9	2.6	+1.5	*G. max*
Ug35505	9.3	19.1	+1.0	*P. trichocarpa*	Ug35988	7.0	2.5	−1.5	*P. trichocarpa*
Ug64791	0.0	17.1	+14.1	*R. australe*	Ug56737	0.6	1.0	+0.8	*P. trichocarpa*
Ug25907	26.2	5.0	−2.4	*A. thaliana*	Ug36012	2.8	0.8	−1.8	*--*
Ug421	13.9	4.9	−1.5	*V. vinifera*	**POD**				
Ug41652	13.3	2.8	−2.2	*A. lyrata*	Ug16495	502.4	158.5	−1.7	*A. lyrata*
Ug9652	5.9	2.5	−1.3	*A. lyrata*	Ug14936	346.9	113.9	−1.6	*G. hirsutum*
Ug42510	1.2	0.5	−1.2	*R. communis*	Ug9314	420.9	71.7	−2.6	*L. chinensis*
Ug38396	1.6	0.0	−10.7	*A. thaliana*	Ug31805	135.2	54.4	−1.3	*T. hispida*
Ug42531	2.9	0.0	−11.5	*P. trichocarpa*	Ug28717	143.6	49.1	−1.5	*L. chinensis*
Ug45519	2.4	0.0	−11.2	*A. thaliana*	Ug21658	127.8	45.9	−1.5	*L. chinensis*
**APX**					Ug24487	110.2	44.3	−1.3	*A. thaliana*
Ug44406	422.1	448.6	+0.1	*R. australe*	Ug8366	216.0	38.5	−2.5	*L. chinensis*
Ug47495	171.9	210.3	+0.3	*T. hispida*	Ug58048	187.9	33.6	−2.5	*O. sativa*
Ug17786	324.5	199.7	−0.7	*N. tabacum*	Ug17264	74.8	25.4	−1.6	*T. hispida*
Ug13095	315.9	183.3	−0.8	*F. ananassa*	Ug26097	75.6	21.7	−1.8	*T. hispida*
Ug57465	336.9	167.6	−1.0	*C. sinensis*	Ug56728	2.9	18.3	+2.6	*T. hispida*
Ug25879	201.8	166.0	−0.3	*A. thaliana*	Ug555	45.7	14.3	−1.7	*B. gymnorhiza*
Ug10954	241.7	143.2	−0.8	*S. nigrum*	Ug33872	37.9	11.6	−1.7	*T. hispida*
Ug26707	48.4	98.9	+1.0	*M. crystallinum*	Ug21205	35.9	9.5	−1.9	*P. trichocarpa*
Ug46803	98.4	43.9	−1.2	*T. hispida*	Ug8210	21.3	7.0	−1.6	*T. hispida*
Ug28905	8.9	3.9	−1.2	*Z. aethiopica*	Ug26203	12.8	5.4	−1.2	*O. sativa*
**MDAR**					Ug23427	22.2	4.8	−2.2	*A. thaliana*
Ug33304	347.9	300.5	−0.2	*P. sativum*	Ug61313	0.0	4.5	+12.1	*O. sativa*
Ug64013	1.3	5.2	+2.0	*A. lyrata*	Ug55297	0.9	4.5	+2.3	*G. hirsutum*
**DHAR**					Ug13312	16.7	4.4	−1.9	*A. thaliana*
Ug42162	275.1	196.6	−0.5	*M. pumila*	Ug63541	1.1	3.6	+1.7	*A. thaliana*
**GR**					Ug3944	15.1	2.9	−2.4	*S. oleracea*
Ug8218	279.8	259.3	−0.1	*R. australe*	Ug47499	5.7	1.4	−2.0	*I. batatas*
Ug3408	310.6	233.2	−0.4	*V. unguiculata*	Ug34920	12.0	1.0	−3.5	*A. thaliana*
**GST**					Ug43924	7.6	1.0	−3.0	*S. oleracea*
Ug48125	250.3	477.2	+0.9	*J. curcas*	Ug43247	2.2	0.5	−2.2	*A. thaliana*
Ug63119	313.4	336.6	+0.1	*Z. mays*	Ug46420	4.1	0.4	−3.3	*S. oleracea*
Ug12484	217.4	220.3	+0.0	*T. androssowii*	Ug47065	3.9	0.4	−3.3	*G. max*
Ug42162	275.1	196.6	−0.5	*M. pumila*	Ug40929	2.5	0.0	−11.3	*A. thaliana*
Ug48024	184.0	195.6	+0.1	*P. trichocarpa*	**PrxR**				
Ug32254	115.3	191.7	+0.7	*T. androssowii*	Ug63275	487.7	279.7	−0.8	*Z. mays*
Ug1025	114.0	155.9	+0.5	*M. pusilla*	Ug61053	226.7	215.5	−0.1	*I. batatas*
Ug20784	117.7	139.8	+0.2	*P. trichocarpa*	**Trx**				
Ug6700	96.3	138.9	+0.5	*E. guineensis*	Ug12493	200.7	353.6	+0.8	*H. brasiliensis*
Ug8174	84.6	127.3	+0.6	*A. thaliana*	Ug17142	502.1	339.8	−0.6	*L. bicolor*
Ug18987	160.0	109.0	−0.6	*E. guineensis*	Ug7940	214.0	177.2	−0.3	*A. thaliana*
Ug62570	203.9	106.5	−0.9	*A. hypogaea*	Ug34328	221.6	169.4	−0.4	*S. tuberosum*
Ug28190	62.6	102.6	+0.7	*A. thaliana*	Ug2658	404.1	157.1	−1.4	*P. trichocarpa*
Ug7332	135.4	64.2	−1.1	*L. bicolor*	Ug21241	18.5	79.8	+2.1	*M. truncatula*
Ug27980	52.8	58.8	+0.2	*P. trichocarpa*	Ug16795	5.7	37.4	+2.7	*N. alata*
Ug27203	41.0	57.6	+0.5	*P. trichocarpa*	Ug37125	15.1	32.9	+1.1	*M. truncatula*
Ug19050	69.7	28.3	−1.3	*N. tabacum*	Ug65122	3.3	30.9	+3.2	*G. max*
Ug4112	38.2	17.2	−1.1	*J. curcas*	Ug14501	43.7	18.6	−1.2	*A. thaliana*
Ug28720	6.3	13.2	+1.1	*G. max*	Ug49635	0.6	18.4	+4.8	*Z. mays*
Ug53783	3.6	12.4	+1.8	*P. trichocarpa*	Ug27430	1.0	10.0	+3.3	*M. truncatula*
Ug14715	11.7	11.7	+0.0	*P. trichocarpa*	Ug58332	18.6	8.8	−1.1	*N. benthamiana*
Ug8711	2.8	10.1	+1.8	*P. trichocarpa*	Ug32797	9.5	4.0	−1.2	*A. thaliana*
Ug8711	2.8	10.1	+1.8	*P. trichocarpa*	Ug61925	0.9	3.1	+1.8	*Z. mays*
Ug58658	1.1	8.1	+2.9	*G. max*	**SOD**				
Ug56087	2.7	5.9	+1.1	*T. androssowii*	Ug23472	332.4	348.8	+0.1	*N. nucifera*
Ug6808	5.3	5.9	+0.1	*P. trichocarpa*	Ug8340	247.0	265.8	+0.1	*T. androssowii*
Ug28520	11.9	5.4	−1.1	*Z. jujuba*	Ug18352	78.3	36.8	−1.1	*M. crystallinum*
Ug30784	2.2	4.9	1.1	*P. trichocarpa*	Ug57351	0.6	1.5	+1.4	*G. max*

*DEG was filtered by using threshold of false discovery rate (FDR) ≤ 0.001 and absolute value of log_2_Ratio ≥ 1. Ug represents unigene. RPKM means RPKM values of unigenes in C21 or T43. “Fold change” equals to log_2_ (T43-RPKM/C21-RPKM). “+” indicates up-regulated transcription and “-” represents down-regulated transcription. Homologous species is that identified from BLAST search of nr database using cut-off E-value of ≤ 10^-5^.

### Experimental validation

**Figure 8:**
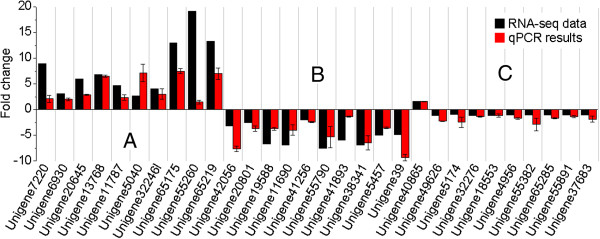
**Expression pattern validation of selected genes by qPCR.** Changes in transcript levels of 30 selected genes as detected by qPCR. **A, B, **and **C** represent 10 up-regulated, 10 down-regulated, and 10 unigenes with changes in transcript abundance of < 2-fold, respectively. X-axis shows -fold change in transcript abundance of unigenes. Black bar indicates transcript abundance changes calculated by the RPKM method. Red bar with associated standard error bar represents relative expression level determined by qPCR using 2^-∆∆CT^ method. Results represent mean standard deviations (± SD) of three experimental replicates.

PCR amplification showed that all qPCR primers produced only single fragments of the expected lengths (155–350 bp, a 100% success rate). The qPCR results for 30 selected unigenes showed general agreement with their transcript abundance changes determined by RNA-seq, suggesting the reliability of the transcriptomic profiling data. However, moderate discrepancies between the expression levels and RPKM values were observed in ten unigenes, i.e., unigene7220, unigene20645, unigene5040, unigene65175, unigene55260, unigene65219, unigene42056, unigene19588, unigene41893 and unigene39 (Figure 8). On the other hand, amplifications of longer sequences (> 500 bp) had a lower success rate. Among the 30 unigenes, 26 unigenes yielded fragments > 500 bp and the other four failed to produce amplification products, yielding a success rate of 86.7%. All positive clones from the validation studies were sequenced by Sanger sequencing, and the results completely confirmed the Illumina results.

## Discussion

### Construction of an informative transcriptome dataset for *R. trigyna*

For many non-model species, there is no background genomic information available for researchers to conduct comprehensive investigations into the genetic mechanisms underlying their unique features. Therefore, the newly developed NGS technique has been used widely to explore genomic solutions to important physiological questions. Gene annotation is an important element of NGS in which biological information is attached to the predicted genes or unigenes. A high proportion of unigenes with high-confidence BLASTX similarity to protein sequences from annotated gene catalogs of other plant species is considered to indicate the integrity of transcript sequences assembled from Illumina short-read data [23]. This assumption was also verified by the results generated in the present study. In *R. trigyna*, 54.27% of 65340 unigenes were annotated by BLAST analysis and functional bioinformatics analyses (e.g., GO, Swiss-Prot, and KEGG). Overall, the top five species with BLAST hits to annotated unigenes were *Arabidopsis thaliana*, *Oryza sativa*, *Arabidopsis lyrata*, *Populus trichocarpa*, and *Vitis vinifera*, species for which the annotations of their genomes are comprehensive and largely accepted. This suggested that the sequences of the *R. trigyna* unigenes generated in the present study were assembled and annotated correctly.

The lengths of the assembled sequences are crucial in determining the level of significance of a BLAST match [20]. Out of all the assembled unigenes, 66.79% had lengths between 300 and 500 bp, among which 37.1% had significant BLAST hits in the public databases. There were 1224 unigenes showing strong homology with the sequences hit in the database (E-value < 1.0E^-50^). Among 21697 unigenes with sequence lengths > 500 bp, 87.0% had significant BLAST scores and 59.2% showed E-values less than 1.0E^-50^. Out of all the unannotated unigenes (28991), 70.65% were longer than 500 bp, demonstrating that the lack of annotation for these unigenes was not because of a shorter sequence length but because of a genuine lack of hits to sequences in the database. Therefore, we can speculate that these unannotated unigenes represent a specific genetic resource of *R. trigyna*, which warrants further investigation.

In this study, we also used other strategies to enhance the effectiveness of the short reads assembly, apart from the use of bioinformatics methods. These included preparation of high-quality RNA samples, removal of dirty raw reads, BLAST assembly of unigenes using multiple databases, and the large sample population for sequencing [20,24]. First, RNA was isolated from sterile *R. trigyna* seedlings to minimize the risk of contamination by foreign RNase. Second, three seedlings were used to extract RNA samples, not only to reduce sample bias, but also to ensure comprehensive coverage of the *R. trigyna* transcriptome. Last, two sequencing libraries (C21 and T43) were merged to generate longer sequences and to increase the sequencing depth. These strategies were an effective way to improve the quality of assembly and annotation of assembled unigenes [23].

In summary, an extensive and diverse expressed gene catalog, representing a large proportion of the transcribed genes in *R. trigyna*, was successfully sampled in the present study. The gene catalog provided a comprehensive understanding of the gene transcription profiles of *R. trigyna*, and laid a solid foundation for further study of salt-tolerance mechanisms and identification of novel genes in this species.

### Transcriptome comparison identified genes related to salt-stress in *R. trigyna*

Gene transcription and/or expression is often compared among different developmental stages, among different plant organs, or among plants under different growth conditions [20-22]. In this study, many genes showing transcriptional changes under salt stress were identified by comparing NaCl-treated seedlings with untreated controls. There were 64694 unigenes showing differences in transcript abundance, and 5032 were defined as DEGs using the thresholds of false discovery rate (FDR) ≤ 0.001 and |log_2_Ratio| ≥ 1. GO clustering analysis suggested the potential biological functions of these DEGs. For example, many DEGs were enriched in GO terms such as “oxidoreductase activity”, “catalytic activity” and “response to stress”. This information will be useful to elucidate salt-tolerance mechanisms and to find new salt-stress-related genes specific to *R. trigyna*. KEGG enrichment analysis identified significantly enriched metabolic pathways or signal transduction pathways involving DEGs. Surprisingly, genes in the phenylpropanoid biosynthesis and flavonoid biosynthesis pathways were transcribed at much high levels under salt stress than in the control in *R. trigyna*, which was not expected from the results of previous studies. This result suggested that genes in these two pathways may play vital roles in resisting oxidation and maintaining membrane integrity.

An outstanding advantage of NGS is the detection of transcripts present in low copy numbers. Among the 5032 identified DEGs, almost half were detected as low-abundance transcripts that were up-regulated after exposure to salt-stress. For example, only 14 reads from C21 could be mapped to unigene24841, encoding flavanone 3-hydroxylase (F3H), compared with 470 reads from T43. Nearly 30-fold up-regulation suggested that this gene may act as a key element in the salt-stress response. Overall, transcriptome comparison analysis provided sufficient information to study the salt-responsive mechanisms and genes related to salt-tolerance related in *R. trigyna*.

### Ion transport genes are important for salt tolerance in *R. trigyna*

Ion transport is a crucial element in response to salt stress in plants. This is particularly true in halophytes [25]. It has been shown that halophytes are able to grow under extreme salinity conditions because of their anatomical and morphological adaptations and/or their avoidance mechanisms [25,26]. *R. trigyna* is a representative recretohalophyte with unique morphological characteristics, such as salt glands and succulent leaves, allowing it to adapt to the salinized conditions of the Ordos desert. Our previous study showed that the salt glands of this species functionally excrete salt ions under normal and salt-stressed conditions. However, the amount of Na^+^ and Cl^-^ excreted significantly increased under salt treatment. In addition, *R. trigyna* shows a strong ability to uptake K^+^ from barren soil. Therefore, it is possible that the ion transport mechanisms and their gene expression patterns in this plant differ from those of other plant species to some extent.

Once Na^+^ is taken up into the cell, ATPases (PM-H^+^-ATPases, V-H^+^-ATPases, and V-H^+^-PPases) are induced to create the driving force for Na^+^ transport. This results in not only extrusion of Na^+^ into the external environment by Na^+^/H^+^ antiporters in the plasma membrane, but also in compartmentalization into vacuoles by tonoplast Na^+^/H^+^ antiporters, which are essential for reestablishing cellular ion homeostasis in salt-stressed plants [25,27-30]. In this study, five V-H^+^-PPases were detected in *R. trigyna*, and were proposed to generate a proton electrochemical gradient. Taking the morphological characteristics of this plant into account, the largest share of the driving force generated by such enzymes is likely to be consumed by the succulent leaves and the salt glands, which include two mesophyll-like collecting cells with obvious vacuoles, and six secretory cells [31]. All mesophyll cells in leaves need V-H^+^-ATPases and V-H^+^-PPases to generate an electrochemical gradient to compartmentalize excess Na^+^ ions into the tonoplast, as a mechanism to cope with physiological drought conditions and ion toxicity [3,25]. When Na^+^ is excreted via secretory cells, both V-H^+^-ATPases and V-H^+^-PPases are required to produce an H^+^ electrochemical gradient to pump Na^+^ into collecting cells. PM-H^+^-ATPases generate sufficient driving force to exchange Na^+^ ions out of the cells. In addition, higher H^+^-pumping activity may be indispensable to maintain the high concentration of K^+^ in salt-stressed *R. trigyna*. The activation of PM-H^+^-ATPases in salinized *Populus euphratica* led to repolarization or hyperpolarization of the plasma membrane and thus decreased NaCl-induced K^+^ loss through depolarization-activated outward rectifying K^+^ channels (DA-KOCs) [32,33]. In *R. trigyna*, 10 out of 17 *RtKOC*s showed high homology with those of *Populus*, demonstrating similar functions of these genes. The analysis of transcripts related to K^+^ uptake showed that the outstanding potassium uptake capacity of *R. trigyna* was probably conferred by enhanced KUP/HAK (potassium transporter) and AKT K^+^ uptake systems, among which the number of up-regulated unigenes (39) far exceeded that of down-regulated unigenes (16) under salt stress. In summary, proton electrochemical gradient-dependent K^+^ maintenance, ions secretion, compartmentalization, and enhanced K^+^ uptake systems may represent important components in reestablishing ion homeostasis in *R. trigyna*.

On the other hand, we detected 12 NHXs that were probably responsible for Na^+^ sequestration. The significantly up-regulated unigene20634 and 5272 showed high homology to the *Citrus reticulata* NHX1 and a *Salsola komarovii* Na^+^/H^+^ antiporter, respectively. The slightly down-regulated unigene8445 and 752 had a highly significant BLAST hit to the Na^+^/H^+^ antiporter of *M. crystallinum* and the NHX1 of *Tetragonia tetragonioides*, respectively. These four unigenes also showed strong homology to the NHX2 of *A. thaliana*, suggesting that these *At*NHX2-like proteins play an important role in avoiding or mitigating the deleterious effects of high Na^+^ levels in the cytosol or in regulating intravacuolar K^+^ and pH, which has been demonstrated in *Arabidopsis*[34]. Our prediction is also supported to some degree by the studies of succulent leaves of the halophytes *Suaeda salsa* and *Halostachys caspica*[35].

In *R. trigyna*, we identified only one moderately expressed and up-regulated PM Na^+^/H^+^ antiporter (SOS1B, unigene798), named *RtSOS1*. The transcript profile of this gene was consistent with those of *ThSOS1* and *PeSOS1*, which remained relatively constant or were slightly up-regulated under salt-tress [36,37]. *Escherichia coli* complementation experiments with *PeNhaD1* rescued salt-exposed bacteria by lowering salt accumulation [38], suggesting that the *P. euphratica* gene *PeNhaD1* also functions as an Na^+^⁄H^+^ antiporter*.* Interestingly, a highly abundant transcript (unigene16859, *RtNha1*) encoding an Nha protein was identified in our dataset. This may play a role in Na^+^ exclusion in *R. trigyna.* At present, it is still unknown how the moderately up-regulated *RtSOS1* achieves effective efflux of excess salt ions under extreme salt-stress, and whether any other gene products are also involved in this process. This requires further investigations in the near future.

### ROS scavenging plays a key role in salt-stress response in *R. trigyna*

Salt stress causes a rapid increase in ROS, including superoxide radicals (·O^2-^), hydrogen peroxide (H_2_O_2_), and hydroxyl radicals (·OH), which perturb cellular redox homeostasis and result in oxidative damage to many cellular components and structures [39,40]. According to our previous studies, ·O^2-^ increased rapidly in *R. trigyna* seedlings treated with 400 mM NaCl, as did the concentrations of other antioxidants, for example, GSH. At the same time, increased activities of antioxidant enzymes (SOD, POD) were also detected. This suggested that *R. trigyna* probably has a similar ROS scavenging system to that of other plant species, and that this system can be enhanced to increase its antioxidative ability. As the first line of defense against oxidative damage, SODs are usually induced by salinity to rapidly dismutate ·O^2-^ into oxygen and H_2_O_2_, which is subsequently removed through various pathways [41]. In this study, 14 SODs were identified. Two of them (unigene23472, unigene8340) were transcribed at high levels, and showed up-regulated transcription under salt stress. Therefore, they were probably associated with enhanced SOD activity and responsible for converting the increased ·O^2-^ into H_2_O_2_. On the other hand, increased GSH could be consumed in the ascorbate-GSH cycle and the GPX cycle [39]. In the ascorbate-GSH cycle, the oxidized ascorbate (i.e., monodehydroascorbate) can be converted into dehydroascorbate (DHA); DHA is then reduced to ascorbate at the expense of GSH, yielding oxidized GSH (i.e., glutathione disulfide). In the GPX pathway, GPX can reduce H_2_O_2_ to the corresponding hydroxyl compounds using GSH. However, to fulfill its function as an antioxidant, GSH must be catalyzed by GSTs, which have GPX activity and can use GSH to reduce organic hydroperoxides of fatty acids and nucleic acids to the corresponding monohydroxy alcohols [42]. Interestingly, in our dataset, there were 35 salt-induced GSTs genes that likely participated in such catalytic processes, especially the 11 *Tau* family GSTs [43]. This finding was similar to the results of other studies on salt-stressed plants [44,45]. Therefore, the interaction between GSH and GST may be an important factor in the ROS scavenging system of *R. trigyna*. In addition, we identified 84 genes encoding components of the PrxR/Trx defense system, which employs a thiol-based catalytic mechanism to reduce H_2_O_2_ and is regenerated using Trxs as electron donors [46].

## Conclusions

This study describes a platform in which publicly available information was matched to the *R. trigyna* transcriptome using NGS technology. The substantially assembled sequences represented a considerable portion of the transcriptome of this plant. Transcriptome comparison identified the transcripts and metabolic pathways that play significant roles in the response to salt stress. These results will prompt studies on the molecular mechanisms of salt resistance, facilitate the analysis of expression profiles of genes related to salt tolerance, and accelerate the discovery of specific stress-resistance genes in *R. trigyna*. The information provided here can also further extend the knowledge on the salt tolerance of halophytes that survive in high sodic stress in areas such as the semi-desert saline areas in Ordos, Inner Mongolia, China.

## Methods

### Plant materials and experimental treatment

Seeds of *R. trigyna* were collected in September 2009 from the Eastern Alxa-Western Ordos area in Inner Mongolia, China. Intact plump seeds were selected, immersed in 10% sodium hypochlorite for 15 min, and then rinsed three times with sterilized double-distilled water. The seeds were germinated in a 150-ml conical flask containing 40 ml MS medium in the dark for 72 h. Germinated seeds were then grown on the same medium at 25°C under 70% relative humidity and a 16-h light/8-h dark photoperiod for 15 d. When seedlings were approximately 10 cm high, they were transferred into a test tube containing 50 ml Â½-strength Hoagland’s medium, and further cultured for another 4 weeks with a change of medium every 2 days. Three healthy seedlings of a similar size were selected for further analysis. Prior to NaCl treatment, leaf samples were collected for RNA extraction from each seedling as controls. The roots were then immersed in Hoagland’s medium containing NaCl. The NaCl concentration was increased stepwise from 100 mM to 400 mM at a rate of 100 mM per step every 8 h. Leaves were collected before each increase in NaCl concentration. Leaves were also collected after the seedlings were treated in 100 mM NaCl for 0.5 h, 1 h, 2 h, 4 h, and 8 h. The leaf samples were immediately snap-frozen in liquid nitrogen and stored at −80°C until analysis.

### RNA preparation

Total RNA was extracted with Plant Plus RNA reagent (DP437, Tiangen, Beijing) according to the manufacturer’s instructions. The extracted RNA was treated with RNase-free DNase I (TaKaRa Bio Inc., Otsu, Shiga, Japan) for 45 min at 37°C to remove residual DNA. RNA quality was evaluated using the Agilent 2100 Bioanalyzer with a minimum RNA integrity number value of 8. In this study, two sequencing libraries were prepared, the C21 library from control samples and the T43 library from salt-stressed samples.

### cDNA library construction and sequencing

Beads with Oligo (dT) (Illumina) were used to isolate poly (A) ^+^ RNA from 20 μg of each RNA pool. Fragmentation buffer (Ambion, Austin, TX, USA) was added to break mRNA into short fragments. First-strand cDNA was synthesized using N6 random hexamers (Illumina Inc., San Diego, CA, USA) and SuperScript® III reverse transcriptase (Invitrogen, Grand Island, NY, USA). The second-strand cDNA was synthesized with DNA polymerase I (Invitrogen, Grand Island, NY, USA). Short fragments were purified with a QIAquick PCR extraction kit (Qiagen, Hilden, Germany) and resolved with EB buffer for end-repair and poly (A) addition, and then linked to sequencing adapters. Agarose gel electrophoresis and PCR amplification were used to select suitable fragments. Two cDNA libraries were constructed and sequenced on the Illumina HiSeq™ 2000 platform. The sequences were base-called and quality-checked by the Illumina data processing pipeline.

### *De novo* assembly of sequences and sequence clustering

The raw reads were filtered to obtain high-quality clean reads prior to assembly. This was performed by removing adaptor sequences, duplicated sequences, reads containing more than 5% “N” (i.e., ambiguous bases in reads), and reads in which more than 50% of the bases showed a Q-value ≤ 5. Transcriptome *de novo* assembly was carried out with the short reads assembly program, SOAPdenovo, with the default settings, except that the K-mer value was 25-mer [47]. The different K-mer sizes were assessed; the 29-mer yielded the best assembly for the desired application and so it was used to construct the de Bruijn graph. Contigs without ambiguous base reads were obtained by conjoining K-mers in an unambiguous path. The reads were then mapped to contigs to construct scaffolds with paired-end information. Paired-end reads were used again for gap filling of scaffolds to obtain unigenes. To evaluate the depth of sequence coverage, all usable reads were realigned to the unigenes with SOAPaligner [48]. To minimize sequence redundancy, the scaffolds were clustered using the GICT [49]. The clustering output was passed to the sequence clustering software CAP3 assembler [50] for multiple alignment and generation of consensus sequences. The sequences that did not reach the threshold set and did not fall into any assembly were assigned as singletons.

### Bioinformatics analysis of sequencing data

To assign predicted gene descriptions for the assembled unigenes, they were aligned against the plant protein dataset of nr, Swiss-Prot/Uniprot protein database, and COG databases, respectively, using BLASTX with a significance threshold of E-value ≤ 10^-5^. To identify the best BLASTX hits from the alignments, putative gene names, ‘CDS’, and predicted proteins of the corresponding assembled sequences were produced. The orientation of sequences was also derived from BLAST annotations. For sequences aside from those obtained from BLAST searches, the EST Scan program was used to predict the ‘CDS’ and its orientation. Functional categorization by GO terms [51] was performed based on the best BLASTX hits from the nr database using BLAST2GO software [52] according to molecular function, biological process, and cellular component ontologies with an E-value threshold of 10^-5^. The data were statistically analyzed using WEGO software [53]. The pathway assignments were carried out by sequence searches against the KEGG database [54], also using the BLASTX algorithm with an E-value threshold of 10^-5^.

### Identification and functional annotation of DEGs

To identify DEGs between the two samples, the transcript abundance of all assembled unigenes were analyzed using the RPKM method [55]. With reference to “the significance of digital gene expression profiles” [56], a rigorous algorithm has been developed to identify DEGs between two samples. The probability of gene A being transcribed equally between two samples was calculated using the following formula:

$$p\left(y|x\right)={\left(\frac{N2}{N1}\right)}^{y}\frac{\left(x+y\right)!}{x!y!{\left(1+\frac{N2}{N1}\right)}^{\left(x+y+1\right)}}$$

where the P-value corresponds to the differential gene expression test; N1 and N2 represent the total clean tag number of C21 and T43 samples, respectively; and x and y denote the tag numbers of the gene of interest present in C21 and T43, respectively.

The FDR is a method to determine the threshold P-value in multiple tests and analysis through manipulating the FDR value. FDR ≤ 0.001 and the absolute value of log_2_Ratio ≥ 1 were used as the threshold to identify DEG. Functional enrichment analysis including GO and KEGG were performed using the following ultra-geometric test to find which DEGs were significantly enriched in GO terms (P-value ≤ 0.05) and metabolic pathways (q-value ≤ 0.05) compared with the whole transcriptome background.

$$Ρ=1-\sum_{i=0}^{m-1}\frac{\left(\begin{array}{c} M\\ i \end{array}\right)\left(\begin{array}{c} N-M\\ n-i \end{array}\right)}{\left(\begin{array}{c} N\\ n \end{array}\right)}$$

### Validation and expression pattern analysis

To experimentally validate the transcriptional abundance results from sequencing and computational analysis, 30 unigenes (10 up-regulated, 10 down-regulated, and 10 unigenes with no significant changes in transcript abundance) were selected for RT-PCR and qPCR analysis. Reverse transcription reactions were performed using SuperScript® III Reverse Transcriptase (Invitrogen, Grand Island, NY, USA) with approximately 5 μg total RNA following the manufacturer’s instructions. Primers for RT-PCR and qPCR were designed using Primer Premier 5 and Beacon Designer 7.0 software, respectively (shown in Additional file 8). *β-Actin* was used as the internal control gene. qPCR was performed on a Qiagen Rotor-gene Q realtime PCR platform (Qiagen, Hilden, Germany) using SYBR-Green real-time PCR mix (TaKaRa Bio Inc., Otsu, Shiga, Japan) to detect transcript abundance. The amplification was achieved by the following PCR protocol: first denaturation 95°C for 30 s, then 40 cycles of denaturation at 95°C for 5 s, annealing and extension at 55°C for 30 s. The relative expression levels of the selected unigenes normalized to *β-Actin* was calculated using 2^-∆∆Ct^ method. All reactions were performed with three replicates, and data were analyzed using Rotor-gene Q series software.

## Abbreviations

NGS: Next-Generation Sequencing; DEG: Differentially Expressed Genes; RPKM: Reads Per Kilobase of exon model per Million mapped reads; FDR: False Discovery Rate; GO: Gene ontology; KEGG: Kyoto Encyclopedia of Genes and Genomes; COG: Clusters of Orthologous Groups; AKT: Potassium Channel; HKT: High-Affinity K^+^ Transporter; CNGC: Cyclic Nucleotide-Gated Channel; KUP: K^+^ Ion Transmembrane Transporter; HAK: High Affinity K^+^ Transporter; CHX: Cation H^+^ Exchanger; KEA: K^+^ Efflux Antiporter; KOC: Outward Rectifying K^+^ Channel; SOS1: Salt Overly Sensitive 1; NHX: Na^+^/H^+^ Exchanger; NhaP: Nhap-Type Na^+^/H^+^ Antiporter; V-ATPase: V-H^+^-ATPase, vacuolar H^+^-ATPase; V-PPase: V-H^+^-PPase, H^+^-Pyrophosphatase; P-ATPase: PM-H^+^-ATPase, Plasma Membrane H^+^-ATPase; ROS: Reactive Oxygen Species; GSH: Glutathione; ·O^2-^: Superoxide Radicals, H_2_O_2_, Hydrogen Peroxide; ·OH: Hydroxyl Radicals; GLR: Glutaredoxin; APX: Ascorbate Peroxidase; MDAR: Monodehydroascorbate Reductase; DHAR: Dehydroascorbate Reductase; GR: Glutathione Reductase; GST: Glutathione S-Transferase; GPX: Glutathione peroxidase; POD: Peroxidases; GLP: Germin-Like Protein; CAT: Catalase; PEX11: Peroxisomal Biogenesis Factor11; Trx: Thioredoxin; PrxR: peroxiredoxin; SOD: Superoxide Dismutase; *A*. *lyrata*: *Arabidopsis lyrata subsp. lyrata*; *A*. *thaliana*: *Arabidopsis thaliana*; *A*. *hypogaea*: *Arachis hypogaea*; *B*. *gymnorhiza*: *Bruguiera gymnorhiza*; *C*. *sinensis*: *Camellia sinensis*; *C*. *chinense*: *Capsicum chinense*; *C*. *quinoa*: *Chenopodium quinoa*; *C*. *limon*: *Citrus limon*; *C*. *unshiu*: *Citrus unshiu*; *C*. *reticulata*: *Citrus reticulata*; *C*. *sativus*: *Cucumis sativus*; *D*. *carota*: *Daucus carota*; *E*. *guineensis*: *Elaeis guineensis*; *E*. *camaldulensis*: *Eucalyptus camaldulensis*; *G*. *max*: *Glycine max*; *G*. *hirsutum*: *Gossypium hirsutum*; *H*. *caspica*: *Halostachys caspica*; *H*. *brasiliensis*: *Hevea brasiliensis*; *H*. *vulgare*: *Hordeum vulgare*; *I*. *batatas*: *Ipomoea batatas*; *I*. *lacteal*: *Iris lacteal*; *J*. *curcas*: *Jatropha curcas*; *K*. *foliatum*: *Kalidium foliatum*; *L*. *bicolor*: *Limonium bicolor*; *L*. *chinensis*: *Litchi chinensis*; *M*. *pumila*: *Malus pumila*; *M*. *pusilla*: *Malva pusilla*; *M*. *truncatula*: *Medicago truncatula*; *M*. *crystallinum*: *Mesembryanthemum crystallinum*; *N*. *nucifera*: *Nelumbo nucifera*; *N*. *alata*: *Nicotiana alata*; *N*. *benthamiana*: *Nicotiana benthamiana*; *N*. *tabacum*: *Nicotiana tabacum*; *O*. *sativa*: *Oryza sativa*; *P. acutifolius*: *Phaseolus acutifolius*; *P*. *sativum*: *Pisum sativum*; *P*. *euphratica*: *Populus euphratica*; *P*. *trichocarpa*: *Populus trichocarpa*; *P*. *juliflora*: *Prosopis juliflora*; *P*. *persica*: *Prunus persica*; *P*. *tenuiflora*: *Puccinellia tenuiflora*; *P*. *communis*: *Pyrus communis*; *R*. *australe*: *Rheum australe*; *R*. *communis*: *Ricinus communis*; *S*. *komarovii*: *Salsola komarovii*; *S*. *lycopersicum*: *Solanum lycopersicum*; *S*. *nigrum*: *Solanum nigrum*; *S*. *tuberosum*: *Solanum tuberosum*; *S*. *oleracea*: *Spinacia oleracea*; *S*. *salsa*: *Suaeda salsa*; *T*. *androssowii*: *Tamarix androssowii*; *T*. *hispida*: *Tamarix hispida*; *T*. *tetragonioides*: *Tetragonia tetragonioides*; *T*. *octandrum*: *Trichostigma octandrum*; *V*. *radiate*: *Vigna radiate*; *V*. *unguiculata*: *Vigna unguiculata*; *V*. *vinifera*: *Vitis vinifera*; *Z*. *aethiopica*: *Zantedeschia aethiopica*; *Z*. *mays*: *Zea mays*; *Z*. *jujuba*: *Ziziphus jujuba*; *Z*. *marina*: *Zostera marina*.

## Competing interests

The authors declare that they have no competing interests.

## Authors’ contributions

ZHD cultivated plants; performed RNA extraction, RT-PCR, and qPCR; analyzed, interpreted, and submitted data; and drafted the manuscript. LLZ and ZQ participated in the design of the study and data analysis. JW and ZG participated in plant management and data submission to the database. SBW participated in analysis and interpretation of data, and critically revised the manuscript. YCW conceived of the study, participated in its design and data interpretation, and revised the manuscript critically. All authors read and approved the final manuscript.

## Supplementary Material

Additional file 1 **Deposited C21-unigenes and TSA accession numbers.** Sequences with no gap and length > 200 bp were selected from sample C21 and submitted to TSA database. Click here for file

Additional file 2 **Deposited T43-unigenes and the TSA accession numbers.** Sequences with no gap and length > 200 bp were selected from sample T43 and submitted to TSA database. Click here for file

Additional file 3 **Functional annotation of all assembled unigenes, including GO, COG, and KEGG analyses. **All-unigene sequences were searched against protein databases (nr, KEGG, COG) using BLASTX (E-value ≤ 10^-5^). Click here for file

Additional file 4 **Summary and functional annotation of identified DEGs. Unigenes with absolute value of |log**_**2**_**Ratio| ≥ 1 and FDR ≤ 0.001 were identified as DEGs.** GO and KEGG analysis of DEGs were based on cut-off E-value of ≤ 10^-5^. Click here for file

Additional file 5 **Summary of DEGs enriched in KEGG pathways.** Pathways and backbone gene numbers are shown in table. The q-value of all these pathways was ≤ 0.05. Click here for file

Additional file 6 Transcripts related to ion transport. Click here for file

Additional file 7 ROS scavenging system-associated transcripts. Click here for file

Additional file 8 Primers used for experimental validation. Click here for file
