# Supplementary material for: Transcriptomic profiling of the salt-stress response in the wild recretohalophyte Reaumuria trigyna
Source: BMC Genomics. 2013 Jan 16;14:29. doi: 10.1186/1471-2164-14-29 (PMC3562145; doi:10.1186/1471-2164-14-29)
Supplement: Additional file 5 — Summary of DEGs enriched in KEGG pathways. Pathways and backbone gene numbers are shown in table. The q-value of all these pathways was ≤ 0.05. [file 1471-2164-14-29-S5.docx]

**KEGG pathway enrichment analysis of DEGs**

| Catalog | Unigene NO. | | Catalog | Unigene NO. | | |
| --- | --- | --- | --- | --- | --- | --- |
| **Phenylpropanoid biosynthesis** | | | **Amino sugar and nucleotide sugar metabolism** | | | |
| caffeoyl-CoA O-methyltransferase | | 3 | chitinase | | 11 | |
| phenylalanine ammonia-lyase | | 3 | 1,4-beta-D-xylan synthase | | 6 | |
| Cytochrome P450 | | 10 | UDP-sugar pyrophosphorylase | | 3 | |
| flavonol 3-O-methyltransferase | | 6 | hexokinase | | 1 | |
| caffeic acid 3-O-methyltransferase | | 11 | phosphomannomutase | | 1 | |
| O-methyltransferase | | 13 | UDP-glucuronate 4-epimerase | | 2 | |
| elicitor-inducible cytochrome P450 | | 2 | sterol-4alpha-carboxylate 3-dehydrogenase | | 1 | |
| carboxypeptidase | | 6 | glucose-1-phosphate adenylyltransferase | | 2 | |
| shikimate O-hydroxycinnamoyltransferase | | 16 | GDPmannose 4,6-dehydratase | | 2 | |
| cinnamoyl-CoA reductase | | 4 | UDPglucose 6-dehydrogenase | | 1 | |
| Bifunctional dihydroflavonol 4-reductase  /flavanone 4-reductase | | 2 | cytochrome-b5 reductase | | 1 | |
| 4-coumarate--CoA ligase | | 4 | **Sulfur metabolism** | | | |
| OPC-8:0 CoA ligase 1 | | 1 | 3'-phosphoadenosine5'-phosphosulfate synthase | | | 2 |
| N-hydroxythioamide S-beta-glucosyltransferase | | 2 | 3'(2'), 5'-bisphosphate nucleotidase | | | 1 |
| cyanohydrin beta-glucosyltransferase | | 2 | adenylylsulfate kinase | | | 1 |
| ferulate-5-hydroxylase | | 2 | desulfoglucosinolate sulfotransferase A/B/C | | | 2 |
| p-coumarate 3-hydroxylase | | 1 | sulfite reductase (ferredoxin) | | | 1 |
| flavonoid 3'-monooxygenase | | 2 | cystathionine beta-lyase | | | 3 |
| UDP-glucosyltransferase | | 7 | cystathionine gamma-synthase | | | 2 |
| sinapate 1-glucosyltransferase | | 4 | **Glycolysis / Gluconeogenesis** | | | |
| **Flavonoid biosynthesis** | | | nuclear factor related to kappa-B-binding protein | | | 1 |
| stilbene synthase | | 1 | phosphoenolpyruvate carboxykinase (ATP) | | | 7 |
| chalcone synthase | | 5 | 6-phosphofructokinase | | | 5 |
| aminocyclopropanecarboxylate oxidase | | 4 | indolepyruvate decarboxylase | | | 2 |
| flavonol synthase | | 23 | phosphoglycerate kinase | | | 1 |
| leucoanthocyanidin dioxygenase | | 4 | hexokinase | | | 2 |
| gibberellin 2-oxidase | | 1 | fructose-bisphosphate aldolase | | | 3 |
| flavonoid 3'-monooxygenase | | 7 | alcohol dehydrogenase | | | 4 |
| cytochrome P450 | | 32 | NAD(P)H-dependent oxidoreductase | | | 3 |
| **Stilbenoid, diarylheptanoid and gingerol biosynthesis** | | | aldehyde dehydrogenase (NAD+) | | | 3 |
| shikimate O-hydroxycinnamoyltransferase | | 15 | tRNA (guanine-N7-)-methyltransferase | | | 1 |
| p-coumarate 3-hydroxylase | | 2 | S-(hydroxymethyl)glutathione dehydrogenase / alcohol dehydrogenase | | | 2 |
| caffeoyl-CoA O-methyltransferase | | 2 | glyceraldehyde 3-phosphate dehydrogenase | | | 4 |
| stilbene synthase | | 1 | pyruvate kinase | | | 1 |
| ferulate-5-hydroxylase | | 1 | **Anthocyanin biosynthesis** | | | |
| Cytochrome P450 | | 65 | flavonol 3-O-glucosyltransferase | | | 2 |
| **Flavone and flavonol biosynthesis** | | | anthocyanidin 3-O-glucosyltransferase | | | 2 |
| flavonol 3-O-methyltransferase | | 14 | flavonol-3-O-glucoside L-rhamnosyltransferase | | | 1 |
| caffeic acid 3-O-methyltransferase | | 22 | N-hydroxythioamide S-beta-glucosyltransferase | | | 2 |
| O-methyltransferase | | 22 | sinapate 1-glucosyltransferase | | | 1 |
| flavonoid 3'-monooxygenase | | 7 | **Tryptophan metabolism** | | | |
| Cytochrome P450 | | 28 | aldehyde dehydrogenase (NAD^+^) | | | 6 |
| ferulate-5-hydroxylase | | 1 | aromatic-L-amino-acid decarboxylase | | | 3 |
| N-hydroxythioamide S-beta-glucosyltransferase | | 1 | indolepyruvate decarboxylase | | | 2 |
| anthocyanidin 3-O-glucosyltransferase | | 2 | N-hydroxythioamide S-beta-glucosyltransferase | | | 2 |
| **Zeatin biosynthesis** | | | sinapate 1-glucosyltransferase | | | 1 |
| adenylate isopentenyltransferase | | 2 | desulfoglucosinolate sulfotransferase A/B/C | | | 19 |
| cytokinin trans-hydroxylase | | 3 | cytochrome P450 | | | 3 |
| cytochrome P450, family 3, subfamily A | | 9 | acetyl-CoA C-acetyltransferase | | | 1 |
| acyl-CoA oxidase | | 1 | 3-hydroxyisobutyryl-CoA hydrolase | | | 2 |
| cis-zeatin O-glucosyltransferase | | 2 | **Peroxisome** | | | |
| N-hydroxythioamide S-beta-glucosyltransferase | | 2 | DNA damage-binding protein | | | 1 |
| cyanohydrin beta-glucosyltransferase | | 2 | peroxin-10 | | | 1 |
| UDP-glucosyltransferase BX9 | | 1 | protein Mpv17 | | | 1 |
| sinapate 1-glucosyltransferase | | 1 | cytokinin trans-hydroxylase | | | 2 |
| **Limonene and pinene degradation** | | | cytochrome P450 | | | 12 |
| aldehyde dehydrogenase (NAD^+^) | | 3 | acyl-CoA oxidase | | | 7 |
| cytochrome P450 | | 62 | fatty acyl-CoA reductase | | | 6 |
| cytokinin trans-hydroxylase | | 1 | Cu/Zn superoxide dismutase | | | 1 |
| flavonoid 3'-monooxygenase | | 2 | 11beta-hydroxysteroid dehydrogenase | | | 1 |
| p-coumarate 3-hydroxylase | | 1 | long-chain acyl-CoA synthetase | | | 7 |
| 3-hydroxyisobutyryl-CoA hydrolase | | 2 | dehydrogenase/reductase SDR family member 4 | | | 2 |
| **Starch and sucrose metabolism** | | | **Monoterpenoid biosynthesis** | | |  |
| pectinesterase | | 15 | casbene synthase | | | 11 |
| polygalacturonase | | 14 | 1,8-cineole synthase | | | 1 |
| UDP-glucuronate 4-epimerase | | 2 | **Linoleic acid metabolism** | | |  |
| 1,4-beta-D-xylan synthase | | 6 | cytokinin trans-hydroxylase | | | 2 |
| UDPglucose 6-dehydrogenase | | 1 | cytochrome P450 | | | 12 |
| trehalose-phosphatase | | 4 | acyl-CoA oxidase | | | 2 |
| putative family 31 glucosidase | | 1 | lipoxygenase | | | 4 |
| alpha-glucosidase | | 5 | NAD(P)H-dependent oxidoreductase | | | 3 |
| sucrose synthase | | 6 | **Nitrogen metabolism** | | | |
| glucan endo-1,3-beta-D-glucosidase | | 12 | carbonic anhydrase | | | 1 |
| beta-glucosidase | | 26 | phenylalanine ammonia-lyase | | | 4 |
| hexokinase | | 1 | L-ascorbate oxidase | | | 6 |
| endoglucanase | | 8 | nitrite reductase | | | 15 |
| glucose-1-phosphate adenylyltransferase | | 2 | cystathionine beta-lyase | | | 3 |
| starch synthase | | 3 | **alpha-Linolenic acid metabolism** | | | |
| starch phosphorylase | | 1 | lipoxygenase | | | 4 |
| beta-amylase | | 4 | hydroperoxide dehydratase | | | 3 |
| alpha-amylase | | 1 | allene oxide cyclase | | | 3 |
| **Phenylalanine metabolism** | | | 12-oxophytodienoic acid reductase | | | 2 |
| peroxidase | | 28 | OPC-8:0 CoA ligase 1 | | | 1 |
| aromatic-L-amino-acid decarboxylase | | 3 | 4-coumarate--CoA ligase | | | 2 |
| aspartate aminotransferase | | 1 | cytokinin trans-hydroxylase | | | 2 |
| phenylalanine ammonia-lyase | | 4 | cytochrome P450 | | | 15 |
| OPC-8:0 CoA ligase 1 | | 1 | acyl-CoA oxidase | | | 7 |
| 4-coumarate--CoA ligase | | 4 | jasmonate O-methyltransferase | | | 1 |
| ferulate-5-hydroxylase | | 1 | **Cysteine and methionine metabolism** | | | |
| p-coumarate 3-hydroxylase | | 1 | cystathionine beta-lyase | | | 5 |
| caffeoyl-CoA O-methyltransferase | | 2 | aspartate aminotransferase | | | 1 |
| **Fatty acid metabolism** | | | cysteine synthase A | | | 2 |
| long-chain acyl-CoA synthetase | | 7 | cystathionine gamma-synthase | | | 5 |
| cytokinin trans-hydroxylase | | 2 | somatic embryogenesis receptor kinase | | | 2 |
| cytochrome P450 | | 12 | UDP-sugar pyrophosphorylase | | | 1 |
| acyl-CoA oxidase | | 7 | DNA (cytosine-5-)-methyltransferase | | | 16 |
| 3-hydroxyisobutyryl-CoA hydrolase | | 2 | splicing factor 3B subunit 4 | | | 1 |
| acetyl-CoA C-acetyltransferase | | 1 | S-adenosylmethionine decarboxylase | | | 1 |
| alcohol dehydrogenase | | 6 | 1-aminocyclopropane-1-carboxylate synthase | | | 1 |
| S-(hydroxymethyl)glutathione dehydrogenase / alcohol dehydrogenase | | 2 | methionine-gamma-lyase | | | 2 |
| aldehyde dehydrogenase (NAD^+^) | | 3 | leucoanthocyanidin dioxygenase | | | 2 |
| **Ascorbate and aldarate metabolism** | | | aminocyclopropanecarboxylate oxidase | | | 6 |
| UDPglucose 6-dehydrogenase | | 1 | flavonol synthase | | | 3 |
| inositol oxygenase | | 1 | **Pentose and glucuronate interconversions** | | | |
| aldehyde dehydrogenase (NAD^+^) | | 3 | pectinesterase | | | 15 |
| D-threo-aldose 1-dehydrogenase | | 1 | pectate lyase | | | 7 |
| L-galactono-1,4-lactone dehydorogenase | | 2 | polygalacturonase | | | 7 |
| L-ascorbate oxidase | | 10 | UDPglucose 6-dehydrogenase | | | 1 |
| nitrite reductase | | 12 | **Benzoxazinoid biosynthesis** | | | |
| L-ascorbate oxidase | | 7 | cyanohydrin beta-glucosyltransferase | | | 3 |
| cytochrome c peroxidase | | 1 | UDP-glucosyltransferase | | | 1 |
| **Diterpenoid biosynthesis** | | | cis-zeatin O-glucosyltransferase | | | 1 |
| ent-kaurene synthase | | 2 | 2-oxoglutarate-dependent dioxygenase | | | 1 |
| casbene synthase | | 13 | flavonol 3-O-methyltransferase | | | 3 |
| 1,8-cineole synthase | | 1 | caffeic acid 3-O-methyltransferase | | | 6 |
| gibberellin 2-oxidase | | 6 | O-methyltransferase | | | 6 |
| Naringenin,2-oxoglutarate 3-dioxygenase | | 1 |  | | |  |
| Flavonol synthase/flavanone 3-hydroxylase | | 1 |  | | |  |
